# Virofree, an Herbal Medicine-Based Formula, Interrupts the Viral Infection of Delta and Omicron Variants of SARS-CoV-2

**DOI:** 10.3389/fphar.2022.905197

**Published:** 2022-07-04

**Authors:** Ly Hien Doan, Li-Wei Chu, Zi-Yi Huang, Anh Thuc Nguyen, Chia-Yin Lee, Chien-Ling Huang, Yu-Fen Chang, Wen-Yu Hsieh, Trang Thi Huyen Nguyen, Chao-Hsiung Lin, Chun-Li Su, Tsung-Hsien Chuang, Jin-Mei Lai, Feng-Sheng Wang, Chia-Jui Yang, Hui-Kang Liu, Yueh-Hsin Ping, Chi-Ying F. Huang

**Affiliations:** ^1^ Institute of Biopharmaceutical Sciences, College of Pharmaceutical Sciences, National Yang Ming Chiao Tung University, Taipei, Taiwan; ^2^ Institute of Biotechnology, Vietnam Academy of Science and Technology, Hanoi, Vietnam; ^3^ Department and Institute of Pharmacology, College of Medicine, National Yang Ming Chiao Tung University, Taipei, Taiwan; ^4^ Program in Molecular Medicine, College of Life Sciences, National Yang Ming Chiao Tung University, Taipei, Taiwan; ^5^ ASUS Intelligent Cloud Services, Taipei, Taiwan; ^6^ Taiwan National Graduate Program in Molecular Medicine, Academia Sinica, National Yang Ming Chiao Tung University, Taipei, Taiwan; ^7^ Immunology Research Center, National Health Research Institutes, Zhunan, Taiwan;; ^8^ LumiSTAR Biotechnology, Inc., Taipei, Taiwan; ^9^ Division of Basic Chinese Medicine, National Research Institute of Chinese Medicine (NRICM), Ministry of Health and Welfare, Taipei, Taiwan; ^10^ Department of Life Sciences and Institute of Genome Sciences, College of Life Sciences, National Yang Ming Chiao Tung University, Taipei, Taiwan; ^11^ Aging and Health Research Center, National Yang Ming Chiao Tung University, Taipei, Taiwan; ^12^ Graduate Program of Nutrition Science, School of Life Science, National Taiwan Normal University, Taipei, Taiwan; ^13^ Department of Life Science, College of Science and Engineering, Fu Jen Catholic University, New Taipei City, Taiwan; ^14^ Department of Chemical Engineering, National Chung Cheng University, Chiayi, Taiwan; ^15^ Department of Internal Medicine, Far Eastern Memorial Hospital, New Taipei City, Taiwan; ^16^ Ph.D. Program in the Clinical Drug Development of Herbal Medicine, Taipei Medical University, Taipei, Taiwan; ^17^ Traditional Herbal Medicine Research Center, Taipei Medical University Hospital, Taipei, Taiwan; ^18^ Institute of Biophotonics, College of Biomedical Science and Engineering, National Yang Ming Chiao Tung University, Taipei, Taiwan; ^19^ Institute of Clinical Medicine, College of Medicine, National Yang Ming Chiao Tung University, Taipei, Taiwan; ^20^ Department of Biotechnology and Laboratory Science in Medicine, School of Biomedical Science and Engineering, National Yang Ming Chiao Tung University, Taipei, Taiwan; ^21^ Department of Biochemistry, School of Medicine, Kaohsiung Medical University, Kaohsiung, Taiwan

**Keywords:** COVID-19, SARS-CoV-2 variants, Omicron variant (SARS-CoV-2), Delta variant (B.1.617.2), ACE2 (angiotensin-converting enzyme 2), miRNA, herbal medicine

## Abstract

Coronavirus disease 2019 (COVID-19) remains a threat with the emergence of new variants, especially Delta and Omicron, without specific effective therapeutic drugs. The infection causes dysregulation of the immune system with a cytokine storm that eventually leads to fatal acute respiratory distress syndrome (ARDS) and further irreversible pulmonary fibrosis. Therefore, the promising way to inhibit infection is to disrupt the binding and fusion between the viral spike and the host ACE2 receptor. A transcriptome-based drug screening platform has been developed for COVID-19 to explore the possibility and potential of the long-established drugs or herbal medicines to reverse the unique genetic signature of COVID-19. *In silico* analysis showed that Virofree, an herbal medicine, reversed the genetic signature of COVID-19 and ARDS. Biochemical validations showed that Virofree could disrupt the binding of wild-type and Delta-variant spike proteins to ACE2 and its syncytial formation via cell-based pseudo-typed viral assays, as well as suppress binding between several variant recombinant spikes to ACE2, especially Delta and Omicron. Additionally, Virofree elevated *miR-148b-5p* levels, inhibited the main protease of SARS-CoV-2 (M^pro^), and reduced LPS-induced TNF-α release. Virofree also prevented cellular iron accumulation leading to ferroptosis which occurs in SARS-CoV-2 patients. Furthermore, Virofree was able to reduce pulmonary fibrosis-related protein expression levels *in vitro*. In conclusion, Virofree was repurposed as a potential herbal medicine to combat COVID-19. This study highlights the inhibitory effect of Virofree on the entry of Delta and Omicron variants of SARS-CoV-2, which have not had any effective treatments during the emergence of the new variants spreading.

## Introduction

Since a novel coronavirus, known as SARS-CoV-2, broke out in late 2019, it has rapidly become a pandemic that the World Health Organization has declared an emergency issue. Clinical features revealed that patients presented with short-term pneumonia (Holshue et al., 2020) and then progressed to multi-organ failure (Mokhtari et al., 2020). In severe patients, coronaviruses induce inflammatory responses and associated lymphopenia (Lucas et al., 2020). The attachment of SARS-CoV-2 spike protein to the ACE2 receptor promotes the viral entry, replication, and new virion formation, then releases the new viral particles which initiate dysregulation of inflammation and cytokine storm (Wang et al., 2020a). Severe COVID-19 patients suffer from cytokine storms, leading to the fatal ARDS with an incidence rate of 41.8%, or irreversible pulmonary fibrosis (Wu et al., 2020). Such uncontrolled systemic hyper-inflammation can be regulated by two primary mediators: ferritin and miRNA. Previous publications have shown that increasing ferritin levels in severe patients (Cheng et al., 2020a) and their miRNAs’ profiles deeply involved the high expression of cytokines (Palanisamy et al., 2012; Gasparello et al., 2021). Although COVID-19 progression and the underlying mechanisms were identified, only 11 emergency treatments and merely 2 approved drugs are used (Center for Drug Evaluation, 2020). All of these current drugs are designed for a single target or a mechanism of action, for example, remdesivir is an anti-virus replication drug while baricitinib and tocilizumab are immunomodulators. However, SARS-CoV-2 induced intricate signaling that blocking a single function caused poor therapeutic efficiency. The emergence of new mutant strains including Alpha (α), Beta (β), Gamma (γ), Delta (δ), and Omicron (ο), which have a 50% higher rate of transmission and infection than the wild Wuhan SARS-CoV-2, has raised concerns about the efficacy of vaccines (Hossain et al., 2021). Therefore, discovering multifunction drugs is highly concerned. In this study, a potential drug was purposed not only to prevent the disease occurrence but also to treat the COVID-19 patient.

To discover complete blockers of the pathological mechanism of COVID-19, the bioinformatics analysis platform proposed drugs capable of inhibiting this disease through multiple biological functions, including virus replication, cytokine storm, and ARDS. By scanning 1.2 thousand-compound profiles in the database (Huang et al., 2019), Virofree was a promising candidate that could reverse SARS-CoV-2 infection signatures through Gene Set Enrichment Analysis (GSEA) (Subramanian et al., 2005a). Some of the ingredients in Virofree include quercetin, hesperidin, genistein, daidzein, and resveratrol, eliciting strong antioxidant and anti-inflammatory effects (Hämäläinen et al., 2007; Parhiz et al., 2015; de Sá Coutinho et al., 2018). Based on unpublished clinical observation, pharmacological properties include prevention and treatment of influenza, secondary treatment for radiotherapy/chemotherapy, reduction in the frequency of asthma attacks, anti-inflammation, tumor cell apoptosis activation, inhibition of cancer metastasis, and damaged gene repair. Furthermore, the biological pathways of Virofree were analyzed through another big data system which is the integration of the databases (KEGG (Kanehisa et al., 2020), Gene Ontology (GO) (Ashburner et al., 2000), and Disease Ontology (DO) (Du et al., 2009; Schriml et al., 2019)). It strongly improved the Virofree connection among ferroptosis, cytokine, miRNA, and ARDS; these mechanisms of action have been frequently reported with COVID-19. Therefore, Virofree could mediate various pathological signals induced by SARS-CoV-2 and provide a comprehensive inhibition to alleviate patients’ syndromes.

Virofree is an outstanding drug that can regulate cytokine secretion and impact other targeting pathways to comprehensively interrupt viral infection. Another target was miRNAs, which played a key role as key modulators in the cellular process. According to reports by Gonzalo et al. (de Gonzalo-Calvo et al., 2021) and Kedzierski et al. (Farr et al., 2021), two teams detected miRNA expression lists in hospitalized COVID-19 patients. There are four common miRNAs in both investigations that regulated the inflammatory response (*miR-150-5p* and *miR-148a-3p*) and viral infection (*miR-92a-3p* and *miR-491-5p*). Based on previous studies, *let-7a* and *miR-148b* were predicted to target the SARS-CoV-2 genome in various regions (Lee et al., 2021). The viral replication can also be inhibited by suppressing the activity of viral M^pro^ which can cleave two overlapping polyproteins pp1a and pp1ab on open reading frame (ORF) 1 to promote more positive-stranded genomic RNA. Therefore, we hypothesized that Virofree could induce levels of *let-7a* and *miR-148b* and inhibit M^pro^, a critical role in the coronavirus life cycle, and stop SARS-CoV-2 replication (Dai et al., 2020). Virofree can simultaneously activate this miRNA subset and inhibit the cytokine storm, which could be a potential therapeutic approach for COVID-19 treatment.

Since the viral replication process requires iron (Michalski et al., 2022), macrophages take up the iron to decrease viral iron bioavailability to inhibit viral replication (Yue et al., 2010). However, excessive iron accumulation in macrophages leads to the potential for oxidatively damaged phospholipids, eventually triggering a specific form of programmed cell death termed ferroptosis (Hinz et al., 2007). Cystine/glutamate antiporter xCT and glutathione peroxidase 4 (GPX4) mediate cellular mechanisms against ferroptosis for glutathione biosynthesis and antioxidant defense (Ader et al., 2021). It is indicated that understanding and targeting macrophages may help increase the efficacy of COVID-19 treatment. The previous description improves the correlation between iron metabolism, the immune system, and ARDS. The last hypothesis function of Virofree altered the stability of iron metabolism and led to the inhibition of the inflammatory response to treat ARDS in COVID-19.

In this study, our drug screening platform for potential SARS-CoV-2 treatments revealed that Virofree could reverse the unique signatures of COVID-19. Furthermore, Virofree was able to block the binding and entry of the SARS-CoV-2 spike, most effectively in Delta and Omicron variants, from multiple variants to ACE2. High levels of ferritin, which were found in the plasma of severe COVID-19 patients, also contributed to ferroptosis and the cytokine storm, leading to fatal ARDS. Virofree could inhibit ferroptosis and TNF-α release amount. Additionally, Virofree can decrease viral replication by suppressing viral M^pro^ and enhancing the level of *miR-148b-5p*. Induction of these targeted miRNAs would downregulate proinflammatory cytokine release. Virofree also showed its ability to improve bleomycin (BLM)-induced ARDS rats’ condition. The reduced fibrosis-related protein expression after treatment suggested a role as an anti-fibrosis agent of Virofree.

## Methods

### Drug Preparation

Virofree, supplied by Geninova Biotech Inc., essentially consists of 250 mg grape seed extract, 180 mg Acerola cherry extract, 160 mg olive leaf extract, 90 mg marigold extract, 80 mg green tea extract, 80 mg pomegranate extract, 80 mg yeast beta-glucan, and 80 mg soya bean extract based on the total weight (1000 mg) of the medicine. The active ingredients are isolated from plant extracts, including quercetin, hesperidin, genistein, daidzein, and resveratrol. The medicine is prepared in capsule form, which is obtained by the method of mixing the components in powder form to obtain a mixture and then encapsulating the mixture.

### Cell Culture

THP-1, a non-adherent human monocytic cell line derived from an acute monocytic leukemia patient (ATCC, #TIB-202), was purchased from the Bioresource Collection and Research Center and cultured at Roswell Park Memorial Institute (RPMI), (Gibco) 1640 supplemented with 10 mM HEPES, 10% fetal bovine serum (FBS), 1% penicillin-streptomycin, and 50 µM β-mercaptoethanol and held in the Petri dish in a 5% carbon dioxide-humidified atmosphere at 37°C. Cells were passaged continuously after 3–4 days. For the experiment, the cell line was used before passage 8.

BEAS-2B, a human normal bronchial epithelial cell line (ATCC, #CRL-9609), was cultured in RPMI medium supplemented with 10% FBS (Invitrogen), 1% PSA, 1% nonessential amino acid, and 2 mM L-glutamate (Invitrogen). Cells were maintained at 37°C with 5% CO_2_ in a cell incubator and passaged every 3–4 days. For the experiment, the cell line was used at the early passage (before passage 6).

LL29 is a human lung fibroblast cell line derived from idiopathic pulmonary fibrosis lung tissue (ATCC, #CCL-134). LL29 cells were cultured in Ham’s F12K medium with 15% FBS (Invitrogen) and 1% PSA. Cells were cultured at 37°C in a 5% CO_2_ atmosphere and trypsinized every 3–4 days. For the experiment, the cell line was used at the early passage (before passage 8).

Baby hamster kidney (BHK)-21 cells, a fibroblast cell line derived from baby hamster kidneys (ATCC, #CCL-10), and Calu-3 cells, a human epithelial lung cell line derived from a patient with lung adenocarcinoma (ATCC, #HTB-55), were cultured in Dulbecco’s Modified Eagle Medium (DMEM, Gibco) supplemented with 10% FBS and 1 × penicillin/streptomycin solution. BHK-21 and Calu-3 cells were incubated at 37°C in a 5% CO_2_ atmosphere and trypsinized every 2 days and 3–4 days, respectively. For the experiment, the cell line was used at the early passage (before passage 8).

### Sulforhodamine B Colorimetric Assay

The sulforhodamine B (SRB) assay is used for cell density determination based on the measurement of cellular protein content. The method described here has been optimized for compound toxicity screening for adherent cells in a 96-well plate. Cells were seeded at 2,000 cells per well for 16–20 h and then treated with different concentrations of different drugs for 24 h. The medium was discarded, and the cells were gently washed twice with phosphate-buffered saline (PBS) and fixed with cold 10% trichloracetic acid (w/v) (SIGMA) at 4°C for 1 h. After fixation, plates were washed twice with water and air-dried. Cells were stained with 100 μl/well of 0.1% (w/v, in 1% acetic acid) SRB solution at room temperature for 1 h and then washed twice with 1% acetic acid (AVANTOR). After air drying, 100 μl of 20 mM Tris-base was added to each well and read at an optical density (OD) of 540 nm.

### Quantitative Reverse Transcription Polymerase Chain Reaction (PCR) Analysis

BEAS-2B cells derived from human normal bronchial epithelium were used to detect biological agents affecting infection mechanisms in the respiratory tract. To evaluate the effect of Virofree on *let-7a* and *miR-148* expression, 1 × 10^6^ BEAS-2B cells were seeded in a 10-cm dish 24 h before drug treatment. Cells were then collected after 24 h of treatment. TRIzol® reagent was used for total RNA extraction, and RNA samples were stored at −80°C. The miRNA levels of *let-7a* and *miR-148b* expression were quantified using quantitative reverse transcription PCR (qRT–PCR) with *U54* as an internal control. Real-time PCR primers were used for amplification, including forward sequences specific for *hsa-let-7a-5p* (5′-GCC​TGA​GGT​AGT​AGG​TTG​TAT​AGT​TA-3′), *hsa-miR148b-5p* (5′-AAG​UUC​UGU​UAU​ACA​CUC​AGG​C-3′), and *U54 (homo)* (5′-GGT​ACC​TAT​TGT​GTT​GAG​TAA​CGG​TGA-3′). qRT–PCR was performed using Phalanx miRNA One Array® profiling (Phalanx Biotech Group).

### Cytokine Determination Assay

The THP-1 cell line was used as a cell model. THP-1 cells were differentiated by 50 ng/ml of phorbol 12-myristate 13-acetate (PMA) (SIGMA; P1585) for 24 h. After washing non-adherent cells with RPMI-free serum, 100 ng/ml of lipopolysaccharide (LPS) (SIGMA; L2654) was used as a stimulator to mimic the inflammatory condition, and treatment of LPS 100 ng/ml alone in differentiated THP-1 cells was considered as the positive control. The cells were treated with the drug with or without the presence of LPS and incubated at 37°C for 6 or 24 h. The cell medium was then collected and stored at −20°C. The level of TNF-α released by treated cells was detected using an enzyme-linked immunosorbent assay (ELISA) kit. The supernatants were analyzed in Nunc MaxiSorp® flat-bottom 96-well plates (Invitrogen, ThermoFisher; #442402) using a human TNF-α uncoated ELISA kit following the manufacturer’s protocol (Invitrogen, Thermofisher; #88-7346). The OD value was measured with the Infinite 200Pro OD reader, using the Tecan i-control program at 450 and 570 nm wavelengths.

### Inhibition of M^pro^ Activity and Determination of the Half-Maximal Inhibitory Concentration (IC_50_)

To determine M^pro^ activity inhibition ability, Virofree was used as an inhibitor in an enzyme-substrate assay in which M^pro^ was cleaved at the cleavage site of the fluorogenic peptide substrate (Abz-TSAVLQSGFRK-Dnp) in PBS. Virofree was incubated at 30°C for 3 min together with a fluorogenic peptide substrate in PBS, followed by protease addition and equilibrated at 30°C for 3 min. The fluorescence emission at 423 nm was detected by excitation at 321 nm using a luminescence spectrometer (PerkinElmer LS50B) (Shi et al., 2020). The IC_50_ value was obtained from the following equation:

$$\text{v}=\frac{{\text{v}}_{0}}{\left(1+{\text{IC}}_{50}^{\text{n}}\right)/{\left[\text{I}\right]}^{\text{n}}}$$

where v is the velocity at different concentrations of the incubated inhibitor [I], and v_0_ is the initial velocity without inhibitor incubation, whereas n is the Hill constant.

### Cell–Cell Fusion

Human lung cancer Calu-3 cells, used as recipient cells, were first seeded in a 12-well plate at 1 × 10^6^ cells per well to form a single layer of cells. BHK-21 cells were seeded at 4 × 10^5^ cells per well in a 6-well plate and transfected with EGFP and spike plasmids (the original Wuhan strain or Delta variant) at a ratio of 1:5 using Lipofectamine 2000. After 24 h, EGFP-spike BHK cells were harvested by adding 1 ml of cell dissociation buffer (5 mM EDTA in PBS) into each well to detach intact cells and resuspended in serum-free DMEM (Gibco). Transfected BHK-21 cells expressing both EGFP and spike genes were used as donor cells, and they were co-cultured in a single layer of Calu-3 cells, used as target cells, for cell–cell contact in the presence or absence of Virofree treatments and incubated at 4°C for 1 h. After 1 h, PBS was used to wash away unbound cells and replaced with a growth medium. Initial images of EGFP-positive cells, representing the binding efficiency, were acquired in five random fields using an inverted fluorescence microscope (Olympus IX70). The binding efficiency of EGFP-positive BHK-21 cells with Calu-3 cells in the control and Virofree-treated groups was quantified by counting the initial number of EGFP-positive BHK-21 cells attached to Calu-3 cells. The number of EGFP cells in the control group was defined as having a binding efficiency of 100%. Therefore, the effect of Virofree on binding efficiency was determined by the percentage of binding efficiency normalized to control. These cells were then treated with the corresponding treatments and then incubated at 37°C for an additional 4 h for wild-type or 2 h for Delta variant, and then randomly selected images of five fields of EGFP-positive cells were acquired to evaluate the fusion efficiency. Syncytial cell formation was calculated by quantifying the expansion area of EGFP-positive cells in these images using ImageJ. The fold change in the EGFP-positive area in the control group from initial to 4 h (wild-type) or 2 h (Delta) was considered as 100% fusion efficiency. The effect of Virofree on syncytia formation was calculated according to the following equation:

$$The\;normalized\;percantage\;\left(\%\right)=\frac{the\;fold\;change\;of\;GFP\;area\;}{the\;fold\;change\;of\;GFP\;area\;in\;control}\times 100$$

### Pseudo-Typed Virus Neutralization Assay

Neutralization assays were performed by incubating wild-type (G-SARS-CoV-2 pseudovirus, WT, LumiSTAR), Delta (G-SARS-CoV2 pseudovirus, B.1.617.2, LumiSTAR), or Omicron pseudoviruses (G-SARS-CoV2 pseudovirus, B.1.1.529, LumiSTAR) with serial dilutions of compounds at the desired concentration in Opti-MEM. HEK-293T cells (1 × 10^4^) stably expressing human ACE2 genes were seeded in 50 µl of Opti-MEM (Gibco) in each well of a black µCLEAR flat-bottom 96-well plate (Greiner Bio-one™), and cells were incubated overnight at 37°C with 5% CO_2_. The next day, each compound was serially diluted three-fold in Opti-MEM and incubated with SARS-CoV-2 pseudo-typed lentivirus at 37°C for 1 h. The tested drug was diluted three times until the lowest concentration was 1 μg/ml. The virus-compound mixture was transferred to the 293T/ACE2 cell plate with a final multiplicity of infection of 0.1. The culture medium was then replaced with fresh DMEM (supplemented with 10% FBS and 100 U/ml penicillin/streptomycin) at 16 h post-infection, and the cells were cultured continuously for another 56 h. After incubating the infected cells at 37°C for 72 h, the GFP fluorescence in the cells was quantified in the ImageXpress Micro Confocal High-Content Imaging System (Molecular Devices).

### Image and IC_50_ Fitting

After incubation at 37°C for 72 h, infected cells were stained with DAPI at 37°C for 20 min. Then, GFP-positive cells and total cell nuclei were detected using an ImageXpress Micro Confocal High-Content Imaging System (Molecular Devices). Raw images (5 × 5 sites, total of 25 sites) were acquired using a 20× water immersion objective lens, followed by processing and stitching using the appropriate settings. Total cells (indicated by nucleus staining) and GFP-positive cells were quantified for each well. All analyses were carried out using the MetaXpress Cell Scoring module, counting positive cells and the total cell numbers at each site. After cell scoring analysis, the raw data were processed by Lumi-Vcal (LumiSTAR custom analysis software). Transduction rates were determined by dividing the GFP-positive cell by the total cell number. Relative transduction rates were obtained by normalizing the infection rates of the drug-treated groups to those of the PBS-treated controls. The inhibition percentage was obtained based on the assumption that the PBS-treated control induced 0% inhibition. The curves of relative inhibition rates versus drug concentration were plotted using Prism 8 (GraphPad). A nonlinear regression method (Muruato et al., 2020) was used to determine the drug concentration at which 50% of GFP (IC_50_) was expressed. Each drug was tested in triplicate. All SARS-CoV-2 pseudovirus neutralization assays were performed at a BSL-2 facility.

### Enzyme-Linked Immunosorbent Assay

An additional experiment was performed using ELISA to evaluate the efficacy of Virofree in interfering with the binding of trimeric SARS-CoV-2 spike protein wild-type (Wuhan strain) or variants (α, β, γ, δ, and ο) and δ variant of the SARS-CoV-2 spike protein RBD domain to biotinylated human ACE2 recombinant protein. First, each well of a 96-well plate was coated with 100 μl of spike protein (500 ng/ml; cat. GTX135972-pro, GeneTex, Taipei, Taiwan) diluted in coating buffer, consisting of sodium carbonate (15 mM) and sodium hydrogen carbonate (35 mM), pH 9.6, at 4°C overnight. The coated plate was then washed thrice with washing buffer consisting of PBS with 0.05% (v/v) Tween‐20 (pH 7.4) and subsequently blocked with 250 μl of blocking buffer consisting of 0.5% (w/v) bovine serum albumin for 1.5 h at 37°C. The plate was washed thrice, then 100 μl of tested drug or inhibitor (10 μg/ml; cat. GTX635791, GeneTex, Taipei, Taiwan) in dilution buffer was added to the plate and incubated for1 h at 37°C;100 μl of biotinylated human ACE2 protein (10 ng/ml; cat. AC2-H82E6; ACRO Biosystems, OX, UK) was added to each well and incubated for another 1 h at 37°C. The binding of spike protein and ACE2 receptor without a drug or inhibitor was considered as a positive control. Before being incubated for1 h at 37°C, the plate was washed three times with a wash buffer, and added with100 μl of streptavidin–HRP conjugate (100 ng/ml; cat. GTX30949, GeneTex, Taipei, Taiwan) in a dilution buffer. The plate was then washed and incubated with 200 μl of TMB substrate per well for 20 min at 37°C under light protection. Subsequently, 50 μl of stop solution was added to terminate the reaction, and the absorbance at 450 nm was detected using a microplate reader (Cytation 5, BioTek, Vermont, USA).

### Western Blot Analysis

The cells were exposed to different treatments for the indicated time, and the cells were lysed on ice with lysis buffer. The cell lysate was cleared by centrifugation at 12,000 g for 10 min. The lysate was resolved by sodium dodecyl sulfate-polyacrylamide gel electrophoresis, and the proteins were transferred to a polyvinylidene fluoride membrane. After blocking with 5% non-fat dry milk in Tris-buffered saline, the membrane was incubated overnight with the desired primary antibody (FPN (Novus Biologicals, NBP1-21502, 1:1000), FTH-1 (Cell Signaling, 4393S, 1:1000), GPX4 (Abcam, ab125066, 1:1000), TFRC (Cell Signaling, 13208s, 1:1000), xCT (Cell Signaling, 17681s, 1:1000), GAPDH (GeneTex, GTX100118, 1:10000), α-SMA (Abcam, ab5694, 1:1000), fibronectin (Santa Cruz, sc-9068, 1:1000), N-cadherin (BD, 610920, 1:1000), and β-actin (GeneTex, GTX109639, 1:10000). Subsequently, the membrane was incubated with an appropriate secondary antibody. Immunoreactive bands were visualized using the enhanced chemiluminescence (ECL) method and captured by a luminescence imaging system (LAS 4000™, Fuji Photo Film Co., Ltd.).

### RNAseq and Data Mining

Total RNA from two concentrations of Virofree and the PBS control sample in BEAS-2B cells was extracted with the RNeasy Mini kit (Qiagen). The mRNA expression level of each sample was detected using next-generation sequencing-RNAseq (Biotools Microbiome Research Center Inc.). The bioinformatics pipeline is outlined in Supplementary Figure S1A. Two different concentrations were used for transcriptomic response profiling, including 66.67 μg/ml (low dose) and 500 μg/ml (high dose). The raw read counts were normalized using “Trimmed Mean of M-values” *via* edgeR (v3.8.1) (Robinson et al., 2010; Maza, 2016), and biologically unduplicated differentially expressed gene (DEG) analysis was performed through the DEGseq package (v1.40.0) using the MARS (MA-plot-based method with random sampling model) method (Wang et al., 2010).

### Gene Set Enrichment Analysis

The GSEA is a computational method that determines whether a pre-defined set of genes show statistically significant differences between two phenotypes (treatment vs. no treatment) (Subramanian et al., 2005b). The goal of GSEA is to determine whether members of a gene set tend to appear at the top (or bottom) of the ranked gene list. The ranked list is based on differential gene expression between two phenotypes. GSEA assigns an enrichment score based on the Kolmogorov–Smirnow statistic for each gene set and then normalizes the score based on its size. A positive score indicates gene set enrichment at the top of the ranked list, whereas a negative score indicates gene set enrichment at the bottom of the ranked list. Finally, based on the normalized enrichment score, a permutation-based false discovery rate is generated to indicate the significance of the enrichment score. GSEA was performed using the C2 and C7 gene set collections from the MSigDB v.7.2. COVID-19 and ARDS-related signatures are sets of genes upregulated or downregulated in disease conditions retrieved from MSigDB and Gene Expression Omnibus (GEO) databases. The GSE76293 microarray retrieved from GEO was analyzed by the limma package. The GSEA analysis was performed using the class ratio for metrics to rank genes, 1000 permutations with gene set permutation types.

### Principal Component Analysis

Gene expression profiles of the two-dose Virofree treatment were adjusted through the sva package to eliminate the batch effect (Leek et al., 2012). PCA analysis was performed *via* prcomp in R.

### Establishing Bleomycin-Induced ARDS Rat Model

Six-week-old male Sprague–Dawley rats were anesthetized with Zoletil 20–40 mg/kg and Xylazine 5–10 mg/kg (intraperitoneal injection) before administering 5 mg bleomycin/250 g body weight (Nippon Kayaku Co., Ltd.) in 200 µl PBS by intratracheal injection and then placed 60^o^ to the left side for 90 min. Animals were randomized to the following treatment: 1) BLM group (*n* = 1) rats were injected intratracheally with 5 mg BLM and sacrificed on day 8. From day 1 to day 7, 0.5 ml of saline was orally administered to the rats twice per day. 2) BLM + Virofree (*n* = 2) rats received intratracheal injection with 5 mg BLM and were sacrificed on day 8. From day 1 to day 7, Virofree with 150 mg/0.5 ml saline was orally administered to the rats twice per day. The dose was decided following an animal equivalent dose calculation based on body surface area (Nair and Jacob, 2016).

### Statistical Analysis

Data are expressed as mean ± SD or SEM. Data were compared to the corresponding control in each experiment by one-way ANOVA, followed by Dunnett’s post-test using Prism 8 (GraphPad). A *p-value* of <0.05 was considered statistically significant.

## Results

### Gene Set Enrichment Analysis Reveals the Reversed Signature of COVID-19 Induced by Virofree

To screen for COVID-19 candidate treatments, transcriptomic response profiles for different drug treatments were analyzed to determine if a given drug could reverse the disease signature gene sets. Signatures of COVID-19, CRS, and ARDS are characterized by genes that are upregulated or downregulated in disease conditions, and reversed signatures refer to genes that are increased/decreased in disease conditions that may be decreased/increased with drug treatment.

The COVID-19 signature was described by Blanco-Melo et al. (2020) as one involving greater upregulation or down-regulation of genes induced by SARS-CoV-2 infection as compared with other respiratory viruses, indicating a unique signature of COVID-19. These gene sets were integrated into the MSigDB database for GSEA analysis. The results suggest that Virofree treatment can reverse the SARS-CoV-2 infection signature in A549 cells, in which genes upregulated by SARS-CoV-2 infection were downregulated (normalized enrichment score (NES) < 0) and downregulated genes were elevated (NES > 0) by the treatment (Supplementary Table S1). Furthermore, it appears that the drug had more effect on cells expressing ACE2.

**FIGURE 1:**
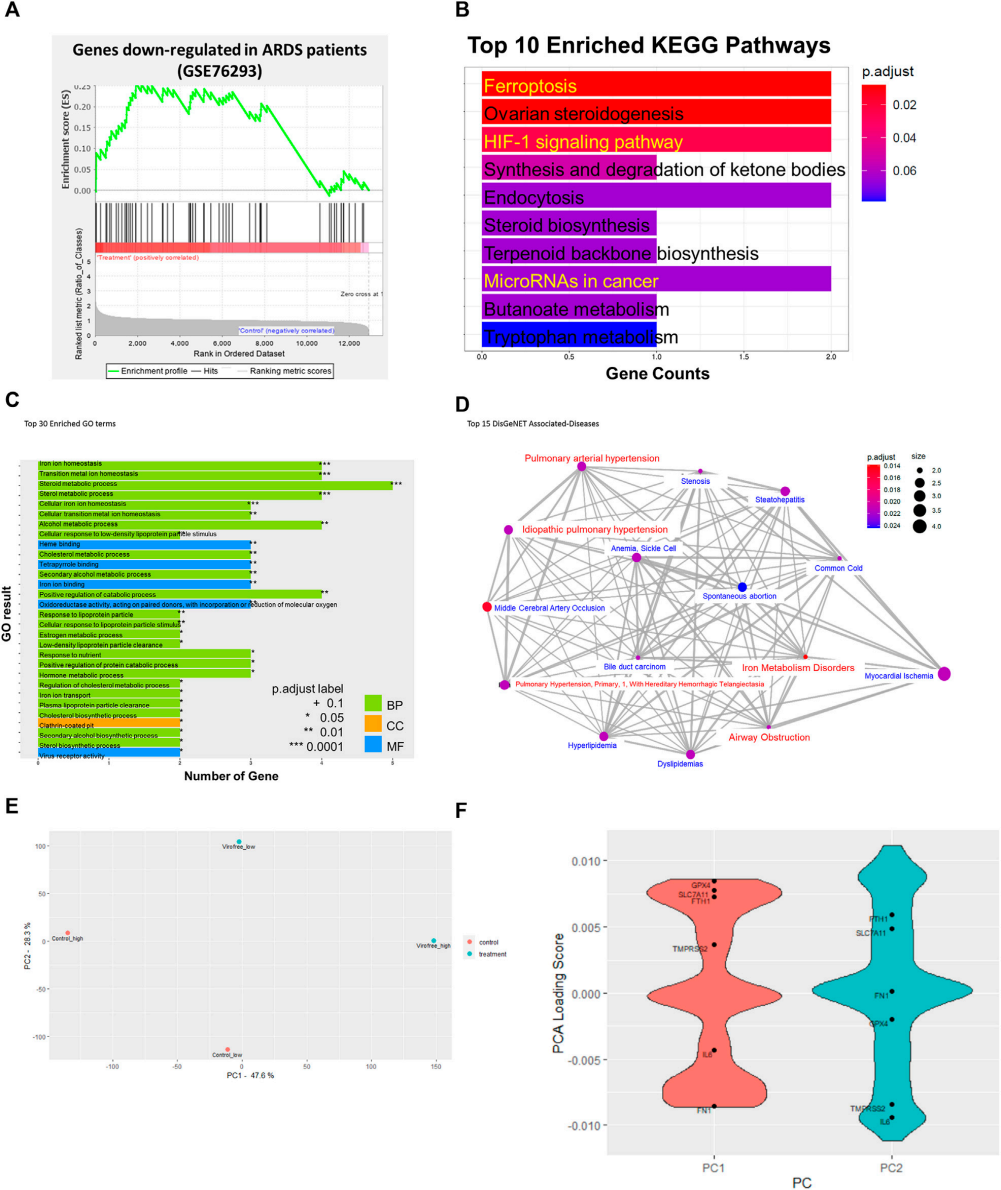
Bioinformatics analysis to identify the underlying mechanism of action of Virofree in the treatment of COVID-19. **(A)** GSEA enrichment plot of gene sets including significantly downregulated genes (log2FC < −1.5, adjusted *p*-value < 0.05) in patients with ARDS. A negative enrichment score (NES) implies an enrichment in the downregulated genes by drug treatment. **(B–D)** bioinformatic analytics for low-dose (66.67 μg/ml) transcriptome profiles of Virofree in BEAS-2B cells. **(B)** top 10 pathways from KEGG enrichment analysis of the DEGs. The color intensity denotes the *p*-value, and the length of the bar indicates the overlapped genes ratio (gene counts) between the input DEGs and the gene set. **(C)** top 30 Gene Ontology (GO) term enrichment. The color indicates the term categories including biological process (BP), cellular component (CC), and molecular function (MF). The length of the bar indicates the overlapped gene number between the DEGs and GO terms. **(D)** associated diseases predicted by the DisGeNET/DO databases. The network illustrated the association among the top 15 diseases, in which the red color highlights the symptoms associated with COVID-19. The statistical results are listed in Supplementary Table S3. **(E, F)** PCA analysis on transcriptomic profiles of low and high doses of Virofree treatment. **(E)** PCA plot of the transcriptomic profiles of low and high doses of Virofree treatment. The gene expression profiles were pre-processed to eliminate the batch effect before PCA analysis. **(F)** loading scores of genes contributing to each principal component (PCs). Positive loading scores indicate a positive correlation between the gene expression and the PC. Negative loading scores indicate a negative correlation between the gene expression and the PC.

GSEA analysis also indicated the effect of Virofree in reducing CRS. There was a decrease of IL-6 signaling in CD4^+^ T cells (NES = −2.41, q-value = 0.003) and an increase in the genes downregulated by interferon-β treatment in bronchial epithelial cells (NES = 1.62, q-value < 0.25), indicating a decreased IL-6 and an increased interferon-β secretion by Virofree treatment (Supplementary Table S1). The effect of Virofree on ARDS was also examined by GSEA analysis using the ARDS signature. The ARDS signature was achieved by significantly up/downregulated genes in blood polymorphonuclear neutrophils (PMNs) from patients with ARDS (GSE76293). A positive NES (NES = 1.87, q-value = 0.01) indicated that Virofree could enhance downregulated genes in patients with ARDS (Figure 1A; Supplementary Table S1).

The GSEA analysis suggests that Virofree could reverse COVID-19 signatures by reducing CRS through inhibition of IL-6 and activation of an interferon-β signaling pathway to reduce ARDS-associated COVID-19.

### The Big Data Analysis Implies That Ferroptosis and miRNA Are Virofree Potential Targets for COVID-19 Treatment

The underlying mechanism of action of Virofree can be revealed through drug treatment gene expression profiling. Transcriptional responses to treatment have profiled the low dose (66.67 μg/ml) of Virofree treatment in BEAS-2B cells. There were six upregulation genes and eight downregulation genes resulting from the low-dose Virofree treatment (Supplementary Table S2B). KEGG enrichment revealed that Virofree functions are related to ferroptosis, HIF-1 signaling pathway, steroid metabolism, and miRNA in cancer (Figure 1B). In addition, GO showed the 30 major biological pathways that mediated iron-ion homeostasis, sterol metabolism process, virus receptor activity, and clathrin-coated pit (Figure 1C). Virofree transcriptomic response profiles were connected to disease signatures via the DisGeNET database, in which the top 10 associated diseases included iron metabolism disorders, airway obstruction, pulmonary arterial hypertension, and idiopathic pulmonary hypertension (Figure 1D). The high Virofree dose (500 μg/ml) demonstrated microRNA regulation from the GO results (Supplementary Figure S2A) whereas the KEGG bar plot showed that treatment was associated with response to the transforming growth factor-beta and response to oxygen levels (Supplementary Figure S2B). Generally, it suggested that Virofree mediated cellular iron-ion homeostasis, miRNA, and viral infection signaling to inhibit COVID-19 symptoms.

Treatments of two different doses of Virofree have been compared by their effects on gene expression levels and associated diseases. Two DEGs were upregulated and four DEGs were downregulated at both drug concentrations; however, *STC2* was the only gene with a significant change in expression levels when the dosage of Virofree was increased (Supplementary Figure S2C). About 700 genes were induced or inhibited by the higher dose of Virofree, and these DEGs were not shown at the lower dose of Virofree, meaning there was no difference between control and treatment. Five commonly associated diseases can be interfered with by low and high doses of Virofree, which are pulmonary arterial hypertension, myocardial ischemia, pulmonary hypertension (primary, 1, with hereditary hemorrhagic telangiectasia), idiopathic pulmonary hypertension, and steatohepatitis, respectively (Supplementary Figure S2D).

Additionally, PCA analysis was performed to compare the transcriptomic response to low and high doses of Virofree (Figures 1E, F). PC1 can mainly describe the transcriptomic profile of high-dose treatment, whereas PC2 can describe the transcriptomic profile of low-dose treatment (Figure 1E). The gene loading scores on each principal component (PC) can demonstrate the distribution of that gene in the PC, or in other words, the expression pattern of that gene in the sample. A positive loading score indicates that the gene expression and a PC are positively correlated. In the case of PC1, since the expression profile of high-dose Virofree treatment is enriched in the positive PC1, genes with positive loading scores in PC1 tend to be upregulated, similar to PC2 and the low dose of Virofree treatment response profile. A negative loading score indicates a negative correlation; therefore, genes with negative loading scores are probably downregulated. A higher magnitude of (either positive or negative) loading scores demonstrates a stronger effect on that PC. The distribution of the loading scores of the two PCs is indicated in Figure 1F. In general, genes related to iron homeostasis including *FTH1* and *SLC7A11* encoding xCT were upregulated in both doses of Virofree, indicating a protective effect against ferroptosis. Interestingly, *IL6* was also downregulated by both doses of Virofree, compared with the control, indicating promising effects of Virofree in blocking the IL-6 signaling pathway to inhibit the cytokine storm. Additionally, fibronectin (*FN1*) expression level is likely to decrease with high-dose Virofree treatment, suggesting the inhibitory effect on fibrosis. The alteration in the expression of these genes was validated in later experiments.

### Virofree Significantly Upregulates Targeter miRNAs to Affect SARS-CoV-2

Since upregulating the expression of *let-7a-5p* and *miR-148b-5p* was likely beneficial in treating COVID-19 (Xie et al., 2021), the effects of Virofree on the expression of these miRNAs were studied. Additionally, the top 100 genes with positive loading scores in the PC1 PCA analysis also show significant enrichment in a gene set containing miRTarBase *let-7a-5p* targets (*p*-value = 0.002; the analysis was performed by CPDB).

**FIGURE 2:**
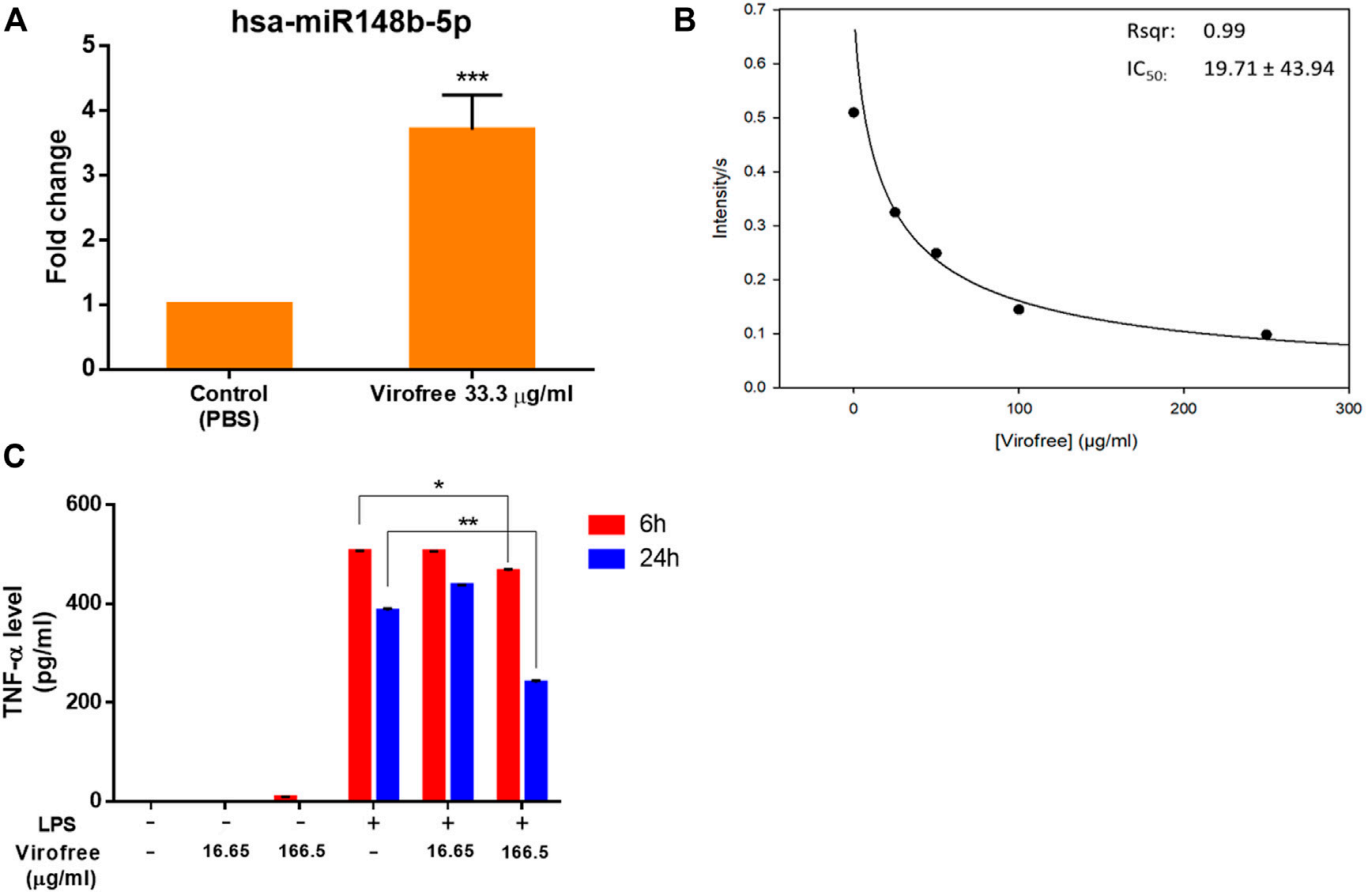
Virofree can potentially reduce viral replication by inducing *miR-148b-5p*, inhibiting M^pro^, and suppressing cytokine storm. **(A)** *miR-148b-5p* expression levels were measured by qRT-PCR after 24 h of treatment with 33.3 μg/ml of Virofree (*n* = 3). All data are presented as means ± SD. **(B)** Virofree IC_50_ of inhibiting recombinant SARS-CoV-2 M^pro^ was determined and showed (µg/ml). **(C)** after treating PMA-differentiated THP-1 cells for 6 or 24 h, the cell medium was collected, and then ELISA was used to measure the amount of cytokine release. Treatment of LPS 100 ng/ml alone in differentiated THP-1 cells was considered as the positive control (*n* = 3). All data are presented as means ± SD. Statistical analysis was carried out with one-way ANOVA. * or **: significantly different from the corresponding control, respectively, with *p* < 0.05 or 0.01.

First, the highest safe dosage of Virofree was determined by treating BEAS-2B cells with different concentrations for 24 h in 96-well plates and evaluating cell viability using the SRB assay. Treated cells maintained a viability rate of more than 80% (Supplementary Figure S3). The qRT-PCR results showed that a low dose of Virofree (33.3 μg/ml) could significantly increase *miR-148b-5p* expression by 3.71 folds (Figure 2A), whereas the high dose of Virofree (333 μg/ml) could increase *let-7a-5p* expression by 1.63 folds (Supplementary Figure S4). These results suggest that Virofree could upregulate the expression of targeted miRNAs in a dose-independent manner.

### Virofree Effectively Suppresses the Cleavage of SARS-CoV-2 M^pro^

Proteolytic cleavage of SARS-CoV-2 polyproteins pp1a and pp1ab by M^pro^ residing on nsp5 releases nsp5–16 and the carboxy (C) terminus of nsp4, whose functions are required for viral replication (V'Kovski et al., 2021). SARS-CoV-2 M^pro^ is a promising target for therapeutic intervention against COVID-19. Therefore, the peptide containing the cleavage site between nsp4 and nsp5 was labeled with a fluorophore (Abz) and its quencher (Dnp) at the N-terminus and C-terminus, respectively, for measuring the protease activity of SARS-CoV-2 M^pro^. The repression of recombinant SARS-CoV-2 M^pro^ activity was examined by Virofree as the inhibitor in a protease activity assay using a fluorogenic probe. The result showed that Virofree could inhibit M^pro^ activity with IC_50_ at 19.71 ± 43.94 μg/ml (Figure 2B), indicating that the drug could suppress SARS-CoV-2 M^pro^ activity.

### Virofree can Inhibit the Cytokine Storm in Macrophages

To quantify the released amount of TNF-α, one of the most abundantly detected cytokines in the plasma acute-phase COVID-19 patients (Choudhary et al., 2021), cell medium was collected at 6 or 24 h Virofree post-treatment to perform ELISA. LPS stimulator treatment alone was considered as the control. Virofree showed its potential in TNF-α suppression in the presence of LPS. The higher dose of Virofree demonstrated a better effect with notable inhibition at both time points, whereas the lower dose could only slightly decrease TNF-α secretion levels at time-point 6 h (Figure 2C). These data suggest that Virofree could partially reduce cytokine storm by inhibiting TNF-α release.

### Virofree Interrupts the Binding of Trimeric SARS-CoV-2 Spike Protein Wild-type (Wuhan Strain) or Variants (α, β, γ, δ, and ο) to Biotinylated Human ACE2 Recombinant Protein

**FIGURE 3:**
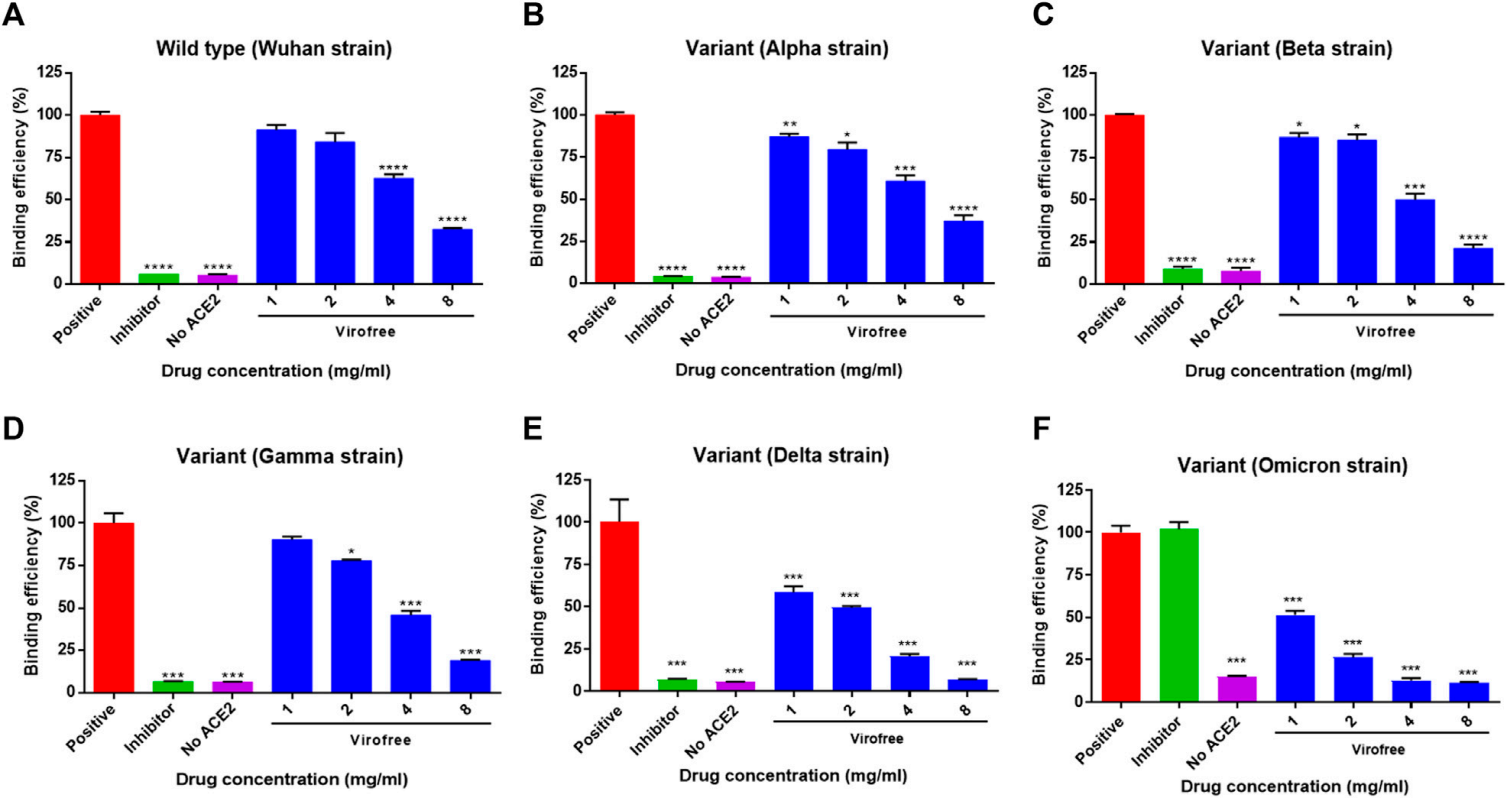
Variant specificity of Virofree against the trimeric spike protein binding to ACE2. **(A–E)** trimeric spike proteins derived from **(A)** wild-type/Wuhan strain, **(B)** variant Alpha, **(C)** variant Beta, **(D)** variant Gamma, **(E)** variant Delta, and **(F)** variant Omicron were used for ELISA-based spike protein and hACE2 binding assays. A positive condition represents the full binding activity of trimeric spike protein on hACE2. The inhibitor used was the wild-type spike RBD antibody (10 μg/ml). Data represent mean ± SEM (*n* = 4). *, **, or *** indicates a significant difference to the corresponding control sample with *p* < 0.05, 0.01, or 0.001, respectively, when compared to the binding efficiency of the positive group correspondingly.

To investigate whether Virofree has a direct inhibitory effect on the binding between SARS-CoV-2 S protein and ACE2, an *in vitro* biochemical binding ELISA assay was conducted using recombinant SARS-CoV-2 S protein and ACE2, and the inhibitory effect of Virofree at doses of 1, 2, 4, and 8 mg/ml was examined. Virofree suppressed the binding efficiency of all five trimeric spike protein strains to ACE2 by approximately 25%–50% (Figures 3A–F). Among them, Virofree appeared to be more effective against the spike proteins of the Delta and Omicron strains than the others (Figures 3E, F). Virofree inhibitory activity against RBD of the Delta variant spike protein was also observed with similar potency against the trimeric form (Supplementary Figure S5). Wild-type spike RBD antibody (10 μg/ml) appeared to be ineffective against the Omicron variant spike protein. These findings suggested that Virofree could directly disrupt the interaction of ACE2 with SARS-CoV-2 S proteins from multiple variants including Omicron.

### Suppression of the Binding of SARS-CoV-2 Spike Protein to ACE2 Receptor and Its Formation of Syncytium of Virofree via Cell-Based Assays

Viral proteins expressed on infected cell membranes can interact with receptors on neighboring naive cells, resulting in cell–cell fusion and syncytia formation (Ortega et al., 2018). Receptor-dependent syncytia formation is triggered by SARS-CoV-2 spike (S) protein in the cell membrane (Cheng et al., 2020b; Buchrieser et al., 2020; Li et al., 2020). Therefore, to further elucidate the anti-SARS-CoV-2 activity of Virofree, the effects of Virofree were measured on both binding efficiency and fusion efficiency between BHK-21 cells, in which wild-type or Delta variant SARS-CoV-2 spike proteins were co-expressed with EGFP and Calu-3 cells with endogenous hACE2 receptor expression. The binding of the BHK-21 cells to Calu-3 cells indicated the interaction of the SARS-CoV-2 S protein with the ACE2 receptor. Furthermore, syncytium formation was caused by membrane fusion between BHK-21 and Calu-3 cells.

**FIGURE 4:**
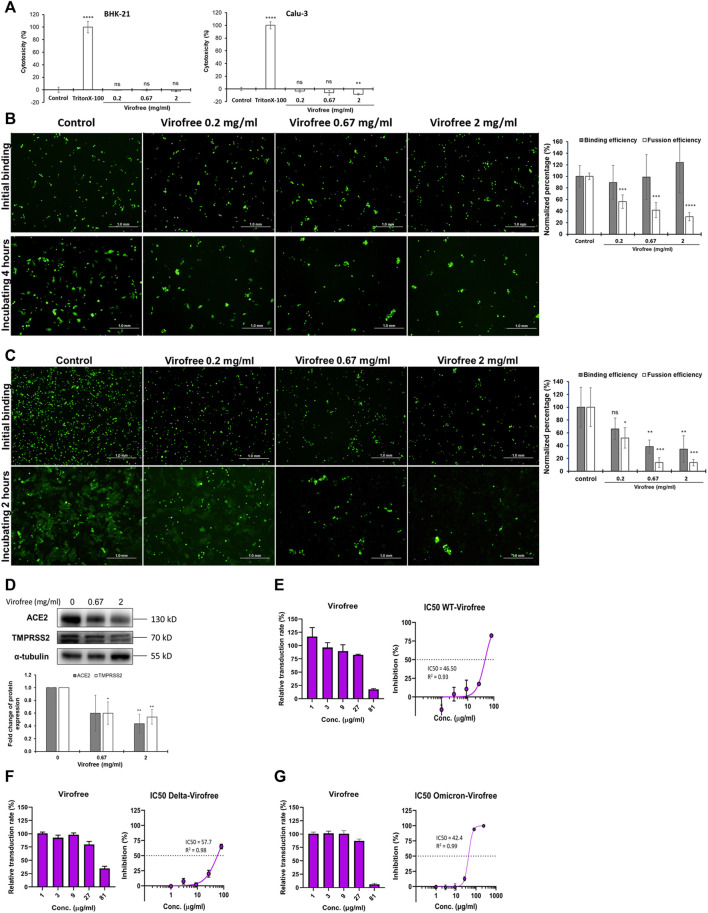
Virofree can interfere with SARS-CoV-2-spike-mediated binding and syncytium formation in cell-based assays. **(A)** cytotoxicity of Virofree was validated by the LDH cytotoxicity assay. BHK-21 and Calu-3 cells were treated with indicated amounts of Virofree, and the cellular cytotoxicity of Virofree was determined by LDH assay. The control group represented no Virofree-treated group, using it as a negative control. The treatment with Triton-100 was serving as a positive control. The data were presented as the mean ± SD; error bars indicated SD. ns indicated non-significant compared to the control group. **p < 0.01 and ****p < 0.0001. **(B, C)** EGFP and wild-type **(B)** or Delta. **(C)** spike co-expressed BHK21 cells were added into Calu-3 cells and incubated at 4°C for 1 h for spike-ACE2 binding. After 4 h (wild-type) or 2 h (Delta) of incubating, the big fluorescence multinucleate cells were formed in the control group, indicating spike-mediated syncytium formation. The histogram depicts the binding and fusion efficiency in each group (*n* = 3). **(D)** Calu-3 cells were treated with 0, 0.67, 2 mg/ml of Virofree for 5 h (*n* = 3). **(E–G)** Virofree can block the infection of pseudovirus expressing SARS-CoV-2 spike protein in ACE2-expressing HEK293T cells in a cell-based neutralizing assay. The inhibitory effect with IC_50_ of Virofree on the entry of wild-type **(E)**, Delta **(F)**, or Omicron. **(G)** SARS-CoV-2 in pseudovirus neutralizing assay (*n* = 3). All data are presented as means ± SD. *p < 0.05, **p < 0.01, ***p < 0.001, and ****p < 0.0001.

To confirm the cytotoxicity of Virofree to BHK-21 and Calu-3 cells, both cells were treated with various concentrations from 0.2 to 2.0 mg/ml of Virofree. The treatment of Triton-100 was serviced as a positive control, and the control group was represented as a non-treatment group. LDH assay clearly showed that Virofree treatment caused no significant amount of cell death, compared to the control group, indicating no cytotoxicity of Virofree to either BHK-21 cells or Calu-3 cells (Figure 4A). In Figure 4B, compared to the control group, there was no significant difference in the binding efficiency of BHK-21 cells with Calu-3 cells among various Virofree treatment groups. After 4 h of incubation, multinucleated cells with expanded green fluorescence signals were formed in the control group, indicating spike-mediated syncytium formation. Furthermore, the wild-type spike-mediated syncytium formation was significantly reduced dose dependently by Virofree treatment. While the SARS-CoV-2 spike Delta variant was expressed in BHK-21 cells, in contrast, Virofree treatment not only caused decreased binding of BHK-21 cells to Calu-3 cells but also significantly inhibited syncytium formation (Figure 4C). The Western blot assay revealed that Virofree treatment dose-dependently reduced ACE2 and TMPRSS2 expression (Figure 4D). The fusion-blocking effect of Virofree was further demonstrated through a cell-based pseudovirus neutralizing assay. The result showed that Virofree could inhibit the entry of pseudoviruses with either wild-type (Figure 4E), Delta (Figure 4F), or Omicron (Figure 4G) of SARS-CoV-2 spike with the IC_50_ at 46.50, 57.71, and 42.40 μg/ml, respectively.

### Virofree Prevents Macrophages From Oxidative-Mediated Toxicity and Ferroptosis

**FIGURE 5:**
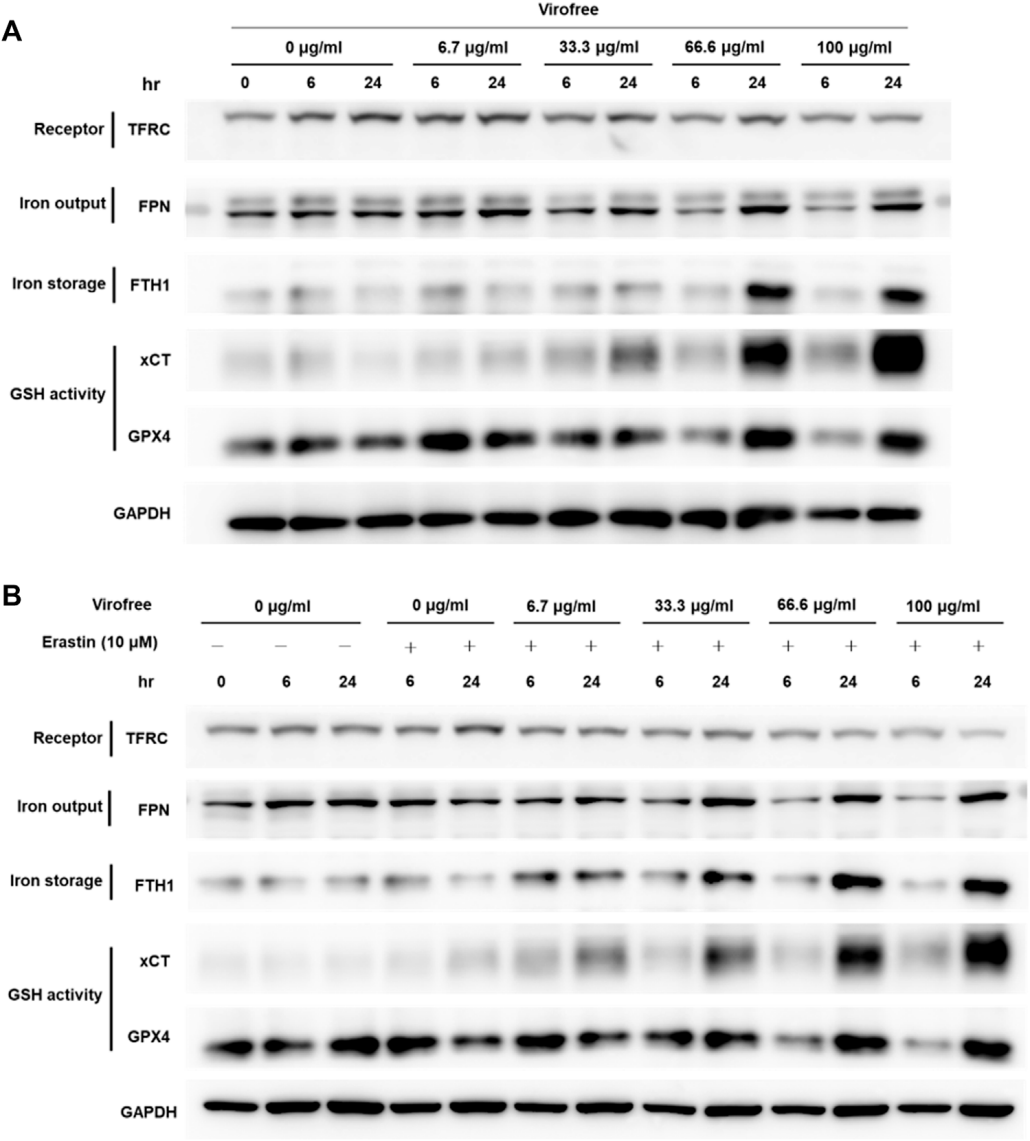
Virofree has the potential to reduce the labile iron pool and protect the cells from ferroptosis in THP-1-derived macrophages. **(A)** THP-1-derived macrophages received treatments of different concentrations of Virofree for 6 or 24 h, respectively. Whole-cell lysates were prepared and subjected to Western blot analysis. GAPDH was used as an internal control (*n* = 3). **(B)** THP-1-derived macrophages received treatments of different concentrations of Virofree in the presence of erastin (10 μM) for 6 or 24 h, respectively. Whole-cell lysates were prepared and subjected to Western blot analysis. GAPDH was used as an internal control (*n* = 3).

To investigate the expression of proteins involved in iron homeostasis in Virofree-treated THP-1-derived macrophages, cells were collected after THP-1 macrophages were incubated with different Virofree concentrations, followed by Western blotting to detect the protein expression levels. The expression of FTH1 and xCT increased dose dependently in THP-1 macrophages after being treated with Virofree (Figure 5A). The results indicate that Virofree might have the potential to enhance the prevention of ferroptosis in macrophages by increasing the expression levels of xCT and FTH1. The protective effects and mechanisms of Virofree against ferroptosis merit further investigation. N-acetyl cysteine (NAC) pretreatment could significantly decrease xCT expression, indicating that elevated ROS levels could induce xCT expression to protect cells (Supplementary Figure S7). To further determine if Virofree could serve as a ferroptosis inhibitor, erastin was used to observe whether Virofree could rescue erastin-induced ferroptosis in cells. Based on Western blot results, Virofree can rescue the downregulated expression levels of ferroportin (FPN) and GPX4, an antioxidant enzyme (Figure 5B), indicating that Virofree could prevent erastin-induced ferroptosis in THP-1 macrophages.

### Virofree Could Inhibit the Expression of TGF-β1-Induced α-SMA and ECM Proteins

**FIGURE 6:**
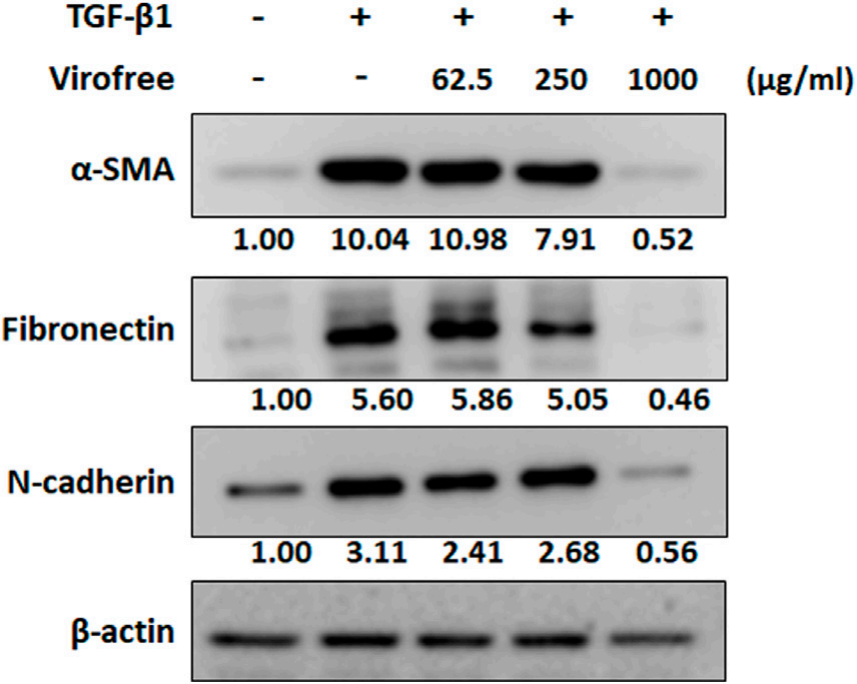
Virofree inhibits TGF-β1-induced α-SMA and ECM protein expression. LL29 cells were co-treated with 2.5 ng/ml TGF-β1 and Virofree at different concentrations for 48 h. Quantification showed a suppressive effect of Virofree on the expression of α-SMA, fibronectin, and N-cadherin expression via Western blot analysis (*n = 3*). All data are presented as means ± SD. *p < 0.05, **p < 0.01, and ***p < 0.001.

COVID-19 infection can lead to ARDS and may potentially cause pulmonary fibrosis with lifelong sequelae (Michalski et al., 2022). TGF-β1 plays an important role in the pathogenesis of fibrotic lung disease by promoting the differentiation of fibroblast cells to myofibroblast cells (Yue et al., 2010) and stimulating the synthesis of ECM components, eventually leading to abnormal fibrosis (Hinz et al., 2007). To confirm the inhibitory effect of Virofree on fibrosis, LL29 cells were treated with TGF-β1 and Virofree for 48 h. The results indicated that Virofree can significantly inhibit TGF-β1-induced α-SMA protein expression in a dose dependent manner. Moreover, the N-cadherin expression level was reduced dramatically by the treatment with Virofree 1,000 μg/ml. In addition, we observed a reduction in TGF-β1-induced fibronectin protein expression level after LL29 cells were treated with Virofree 1,000 μg/ml (Figure 6; Supplementary Figure S9). These data indicate that Virofree had an inhibitory effect on TGF-β1-induced fibrosis.

**FIGURE 7:**
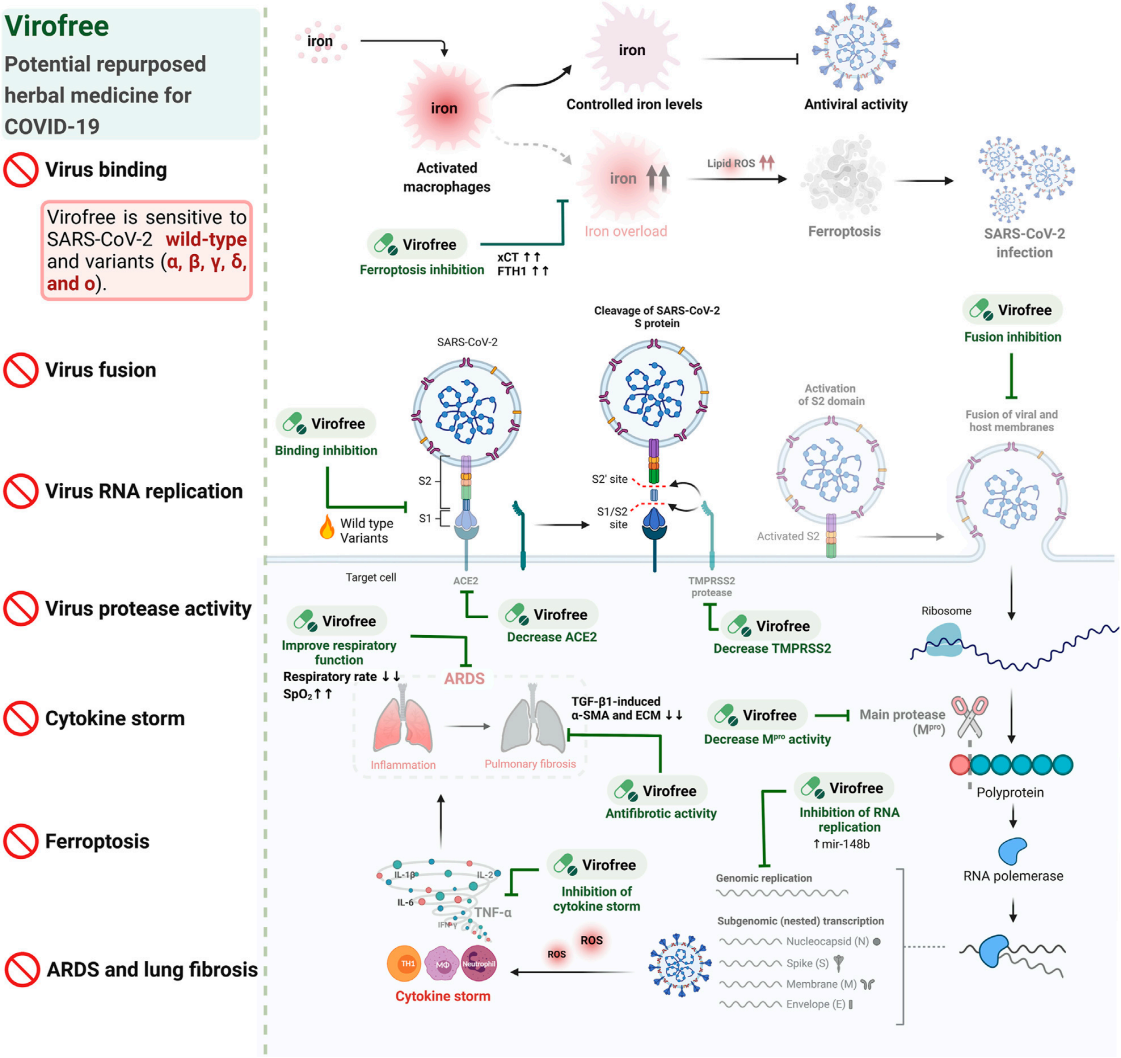
Virofree is a candidate therapeutic treatment for COVID-19.

In summary, Virofree exhibited multiple functions for targeting SARS-CoV-2. Virofree (1) blocked virus entry by interrupting protein binding between the different subtypes of spike and ACE2, as well as reducing ACE2 and TMPRSS2 protein expression, (2) decreased viral replication by increasing *miR-148b* level and inhibiting M^pro^ activity, (3) contributed to cytokine storm reduction by repressing LPS-induced TNF-α production, (4) prevented ferroptosis of THP-1 macrophages, and (5) reduced TGF-β1-induced fibrosis *in vitro*. Our study reveals that Virofree is a promising herbal medicine for further COVID-19 preclinical and clinical studies on the emergence of the Delta and Omicron variants and their transmission (Figure 7).

The invasion of the virus into host cells through the replication process may result in cytokine storm and multi-organ failure. Iron uptaking is one of the symptoms observed in COVID-19 patients, which may lead to ferroptosis promoting viral infection. In our study, Virofree could serve as a ferroptosis inhibitor and antiviral agent by suppressing viral entry and fusion of wild-type and multiple variants of SARS-CoV-2 spike via reducing ACE2 and TMPRSS2 protein expression, inhibiting viral protease activity, increasing *mir-148b*, targeting cytokine storm, as well as downregulating fibrosis proteins *in vitro.* The fainted patterns illustrate the reduction or inhibition. Created with BioRender.com.

## Discussion

As of 30 September 2021, the FDA has announced more than 640 drug development programs in the planning stages, 470 more reviewed trials, 11 treatments authorized for emergency use, and only 1 approved treatment. Remdesivir is the first and only approved drug that targets antiviral replication. Several larger cohorts captured observational data on remdesivir outcomes, reporting a lower risk of death and clinical improvement in the early illness stage (Ader et al., 2021; Mozaffari et al., 2021) but no overall mortality benefit for all hospitalized patients and no significant different response in patients with severe COVID-19 (Goldman et al., 2020). Other therapeutic strategies were immunomodulators (baricitinib (JAK inhibitor) and tocilizumab (IL-6 receptor blocker)) and spike protein monoclonal antibodies (such as casirivimab/imdevimab and bamlanivimab/etesevimab) that showed limitations in the effectiveness of treatment for some patients (Cao et al., 2020). Not to mention ongoing clinical trials, lopinavir-ritonavir, intravenous ribavirin, umifenovir, corticosteroids, or interferon-alpha-2b did not report the consistent clinical benefit (Wong et al., 2021). Recently, Omicron has emerged in a COVID-19 weary world, which has higher transmissibility and viral binding affinity. Importantly, the effects of most of the remaining Omicron mutations are unknown, leading to a high level of uncertainty about the effects of the entire combination of deletions and mutations on viral behavior and susceptibility to natural and vaccine-mediated immunity. The increase in reinfection cases is consistent with the immune-escape mutations present in Omicron (Karim and Karim, 2021). Until now, only nirmatrelvir is effective against Omicron *in vitro* (Greasley et al., 2022). According to previous publications, the pathological process of SARS-CoV-2 infection starts with the binding of viral spike protein and the human ACE2 receptor, eventually entering cells, replicating, and forming new. The protease is then cleaved into two small polypeptide fragments (*p*p1a and *p*p1b) for viral replication and transcription (Huff et al., 2021; Ratre et al., 2021). Proteolysis then packages the components for a new virion by the coronavirus main protease (M^pro^) and releases the functional viral polypeptide (Jin et al., 2020; Huff et al., 2021). These latter factors trigger the cytokine level and dysregulate iron concentration, leading to ferroptosis and fetal ARDS syndrome. In general, there was more than one mechanism to control the pathogenesis of SARS-CoV-2, and the current treatment targeted a single biological function that would ignore other signals of the virus’ ferocity. Therefore, it is necessary to discover a new drug with multiple functions that completely interrupts SARS-CoV-2 infection.

Big data analysis provides a systematic and comprehensive approach to drug development by linking drug response profiles with disease signature and mechanism of action. Large-scale screening of compound reference profiles against the COVID-19 signature via GSEA suggested that Virofree, a safe and healthy herbal medicine, was one of the potential candidates (Figure 1A). The enrichment analysis revealed that Virofree could aim at multiple targets for COVID-19 treatment. The top-ranking KEGG and GO-enriched pathways included iron-ion homeostasis, iron-ion binding, and ferroptosis (Figures 1B–D). COVID-19 patients had abnormally lower serum iron levels (<7.8 μmol/L), and hyperferritinemia in several prevalence studies (Zhao et al., 2020; Hippchen et al., 2020), in which iron dysregulation and overload stimulated the generation of ROS to damage organs, leads to inflammatory activation. The HIF-1 (hypoxia-inducible factor-1α) signaling pathway, which plays an important role in virus infection and proinflammatory responses (Tian et al., 2021; Serebrovska et al., 2020), was significantly enriched in gene expression profiles of Virofree treatment (Figure 1B), suggesting effects of the drug on this pathway to suppress IL-6 production, which is consistent with PCA loading scores (Figure 1F), and eventually inhibit the cytokine storm. GO enrichment indicated that Virofree can mediate clathrin-coated pit (Alfarouk et al., 2021) and virus receptor activity, which are related to the virus–human entrance interaction (Pandey et al., 2021). In contrast, the disease association via DO shows many COVID-19 symptoms or diseases in the upper range of Virofree treatment, including iron metabolism disorders, pulmonary arterial hypertension, idiopathic pulmonary hypertension, airway obstruction, and the common cold. In general, Virofree can regulate the comprehensive pathological mechanisms of SARS-CoV-2 through different mechanisms, and our results agree with previous studies and clinical evidence. Recently, different SARS-CoV-2 variants were explored that made the treatment more difficult and complex. Virofree response profile and these variant signatures can be analyzed further to determine the suitable treatment regimen.

Experimental validation proved that Virofree increased the expressions of *let-7a-5p* and *miR-148b-5p* in BEAS-2B cell lines (normal bronchial epithelial cells). The *let-7* family attenuates the diversity of viral infection, for example, the virulence of influenza viruses interruption, which associates pneumonia and ARDS symptoms (Xie et al., 2021). Sardar et al. (Sardar et al., 2020) mentioned that *let-7a* targeted nonstructural proteins of SARS-CoV-2, and Chauhan et al. (Chauhan et al., 2021) reported that other family members can regulate TMPRSS2. As the immunomodulator, *let-7a* could bind to the TLR4 mRNA strand to block downstream MyD88 and NF-κB signaling including IL-6, which drives innate immunity and proinflammatory response (TNF-α activation) (Chen et al., 2007). Meanwhile, *miR-148a* plays another modulator in the proinflammation, a target miRNA of TLR3, which also regulates the IKK/NF-κB pathway, MyD88, and cytokines (IFN-α, IFN-β, IL-6, and IL-8) (Guo et al., 2020). Some studies predict that *miR-148a* is a high potential candidate for targeting the SARS-CoV-2 genome through bioinformatics (Saçar Demirci and Adan, 2020; Milenkovic et al., 2021). Although the regulation among *miR-148b-5p, let-7a-5p*, and cytokine production was not elaborate, Virofree stimulated these two miRNAs’ expression levels (Figure 2A; Supplementary Figure S4) and reduced TNF-α (Figure 2C). In the aforementioned evidence, Virofree demonstrated the multiple functions (miRNAs and cytokines) to suppress the SARS-CoV-2 pathological mechanism.

Furthermore, Virofree showed two additional functional properties including inhibition of M^pro^ to stop virus replication and interruption of SARS-CoV-2 S protein binding to ACE2 to block viral entry. The SARS-CoV-2 replica gene encodes two overlapping polyproteins, pp1a and pp1ab, that are required for viral replication and transcription. Functional polypeptides are released from polyproteins through the protein hydrolysis processes, primarily by M^pro^ (also known as 3C-like protease). M^pro^ digests polyproteins at 11 conserved sites. The functional importance of M^pro^ in the viral life cycle and the absence of closely related homologs in human make (Hegyi and Ziebuhr, 2002; Pillaiyar et al., 2016; Wang et al., 2020b), M^pro^ as a tempting target for antiviral drug design. The enzymatic assay showed that Virofree could inhibit M^pro^ activity at IC_50_ of 19.71 ± 43.94 μg/ml (Figure 2B).

The ELISA-based trimeric spike-ACE2 binding assay demonstrated that Virofree can block binding between ACE2 and several spike proteins, including wild-type, α, β, γ, δ, and ο variants (Figure 3). Additionally, the result also suggested that the inhibitory effect of Virofree against the spike protein might be related to the RBD of the spike protein. Among these variant spike proteins, the Delta and Omicron variants showed the most sensitive inhibition by Virofree (Figures 3E, F). The inhibitory activity of the wild-type spike RBD antibody against the Omicron variant spike protein was not observed. This observation is consistent with the recent report showing that COVID-vaccine protection is weaker against Omicron (Callaway, 2021). As a result, Virofree appears to show a broader spectrum against spike proteins derived from various variants compared with the target-specific RBD antibody. The results of the cell–cell fusion assay showed that the binding of Delta spike and ACE2 could be inhibited by Virofree treatment (Figure 4C), which is in line with the results of the ELISA-based trimeric spike-ACE2 binding assay (Figure 3E). Furthermore, SARS-CoV-2 spike-mediated syncytium formation could mean that virus-induced cell fusion facilitates viral genome delivery to neighboring cells (Lin et al., 2021). While examining the histopathologic lung sections from deceased COVID-19 patients, the prevailing existence of syncytia cells containing 2–20 nuclei was observed, indicating the correlation between syncytia formation and severe pathogenesis. Based on these observations, using spike-mediated cell fusion to validate drugs against SARS-CoV-2 infection could be a potent strategy (Braga et al., 2021). The time-course experiments were conducted in both wild-type and Delta spike proteins (Supplementary Figure S8). Compared with the wild-type, the Delta strain showed higher binding ability at the initial binding step and faster fusion kinetics at the following time points. The Delta variant reached almost complete fusion within 2 h; in contrast, the percentage of cell fusion in the wild-type remained relatively low. Therefore, we have illustrated the fusion results of the Delta variants at the time point of 2 hours instead of 4 hours (Figure 4B). The results of the cell–cell fusion assay clarified the suppression of spike-mediated cell–cell fusion and pseudovirus infection by Virofree treatment (Figures 4B, C, E), implying that Virofree may not only interrupt the binding of variant spikes and ACE2 but also affect host protease-involved SARS-CoV-2 membrane fusion. In both ELISA and cell–cell fusion assay, Delta variants displayed higher sensitivity to Virofree treatment, compared with wild-type S protein. Because the spike-mediated syncytium formation of the Delta strain was stronger than that of other SARS-CoV-2 variants (Rajah et al., 2021), Virofree which can inhibit the binding of Delta spike and ACE2 sensitively would become a potent drug against Delta variant infection. Additionally, Western blot assay revealed that Virofree treatment could reduce TMPRSS2 expression in a dose dependent manner (Figure 4D), suggesting that Virofree inhibition of SARS-CoV-2 infection could be due to decreased expression of TMPRSS2, which can cleave the SARS-CoV-2 S protein to trigger membrane fusion of SARS-CoV-2 and host cells to facilitate virus entry (Hoffmann et al., 2020). Taken together, these findings indicated that Virofree could inhibit viral binding and entry through two different mechanisms: the inhibition of ACE2 and TMPRSS2 expression and the direct interruption of SARS-CoV-2 S protein binding to ACE2. Furthermore, recent studies have also revealed the pathological role of spike proteins not only in promoting pulmonary vascular remodeling and vascular endothelial cell dysfunction but also in causing pulmonary arterial hypertension (Suzuki and Gychka, 2021). ACE2 acts as a receptor in response to spike protein stimulation (Suresh and Suzuki, 2021). Therefore, inhibition of spike protein binding to ACE2 by Virofree may also contribute to lung protection in addition to preventing viral infection. In addition, as shown in Figure 2, inhibition of viral replication and cytokine storm appear to be intracellular events. Moreover, the interruption of spike protein/ACE2 interaction (Figure 3) and attenuation of viral infection (Figure 4) are extracellular events. We speculate that this difference in targeting components might be explained by differences in required dosages for inhibiting viral replication, cytokine storm, and attenuating viral infection.

The inflammatory condition of COVID-19 patients is also associated with iron metabolism dysregulation (Chifman et al., 2014). However, excess intracellular iron can promote ROS production. System xc^–^, the cystine/glutamate antiporter (a transmembrane protein complex containing SLC7A11 and SLC3A2 subunits), is increased for glutathione synthesis to inhibit cellular ROS production (Sato et al., 2018). Intracellular iron, which is kept in relative balance by uptake and metabolism, can be exported out of the cell through FPN or be stored in Fe (III) via ferritin to prevent oxidative stress caused by excess free iron within cells. SARS-CoV-2 infection promotes iron accumulation in cells, which promotes ferroptosis and ultimately leads to organ failure. There is also evidence that SARS-CoV-2 can infect macrophages which involve in iron metabolism and inflammatory response (Soares and Hamza, 2016) through certain mechanisms (Abassi et al., 2020), indicating macrophages as an attractive therapeutic target. The experimental results showed that Virofree could enhance the expression levels of FTH1 and xCT (SLC7A11) in macrophages and could reverse the downregulation of FPN and GPX4 expression levels caused by erastin (ferroptosis inducer) treatment (Shibata et al., 2019), demonstrating the potential of Virofree to prevent macrophages from ferroptosis. Another finding is that Virofree downregulated TFRC expression levels in cells from bioinformatics analysis and experimental results (Figure 5). It was also reported in a study that TFRC directly interacts with the SARS-CoV-2 spike protein to mediate viral entry, which also suggests that TFRC is an alternative receptor for SARS-CoV-2 cell entry (Tang et al., 2020). Currently, no ACE2 inhibitors have been beneficial to COVID-19 patients, and this highlights TFRC as a promising anti-COVID-19 target.

High serum levels of inflammatory cytokines and elevated ferritin in patients with severe COVID-19 correlate with disease severity and inflammation and iron-ion metabolic derangement (Del Valle et al., 2020; Yang and Lai, 2020). The main symptoms of severe SARS-CoV-2 infection include severe pneumonia and ARDS. Lung damages, including extensive interstitial and alveolar inflammatory infiltrates, alveolar septal thickening, vascular congestion, and pulmonary edema, were found after SARS-CoV-2 infection, which in some patients may be associated with the development of irreversible pulmonary fibrosis (Yang et al., 2020). A previous cohort study also indicated that almost 87% of COVID-19 patients had pulmonary fibrosis after SARS-CoV-2 infection (Suzuki et al., 2021). TGF-β1 plays an important role in the pathogenesis of fibrotic lung disease, by promoting fibroblast cells differentiation to myofibroblast cells (Yue et al., 2010) and stimulating the synthesis of ECM components, eventually leading to abnormal fibrosis (Hinz et al., 2007). Treatment of Virofree on TGF-β1-induced fibrosis LL29 cells for 48 h indicated that Virofree can significantly inhibit TGF-β1-induced α-SMA and N-cadherin protein expression and notably reduce fibronectin (Figure 6; Supplementary Figure S9).

For a better simulation of the patients’ condition, a BLM-induced ARDS rat model was used to investigate the effects of Virofree. Our preliminary observation showed that Virofree treatment improved the condition of ARDS rats by increasing arterial oxygen saturation and reducing breathing rate (Supplementary Figure S10). Since a high level of TNF-α is involved in the progression of ARDS, anti-TNF-α has been considered one of the pivotal therapies for this disease (Malaviya et al., 2017). That Virofree was able to decrease TNF-α secretion amount, fibrosis-related protein expression *in vitro* (Figure 6; Supplementary Figure S9), and improve physiological indexes of ARDS rats in the preliminary *in vivo*, and results suggest that Virofree is a potential anti-COVID-19 and anti-fibrosis treatment. Further investigations with additional rats are needed to confirm its mechanisms in treating ARDS.

Recently, several therapeutic drugs for treating COVID-19 have been approved for Emergency Use Authorization, such as nirmatrelvir/ritonavir (Pfizer) or molnupiravir (Merck). However, they can only target viral M^pro^ or disrupt RNA replication, respectively. Due to the emergence of mutant strains which grow faster than the speed of drug development, it is necessary to discover and advance broad-spectrum drugs to target multiple risk factors caused by SARS-CoV-2.

Proinflammatory cytokines induce the formation of large amounts of nitric oxide (NO) by inducible nitric oxide synthase (iNOS), and compounds that inhibit NO production have anti-inflammatory effects. Some of the ingredients in Virofree include flavonoids, such as quercetin, hesperidin, genistein, daidzein, and resveratrol, which elicit strong antioxidant and anti-inflammatory effects (Hämäläinen et al., 2007; Parhiz et al., 2015; de Sá Coutinho et al., 2018). It has been reported that quercetin, genistein, and daidzein can inhibit iNOS protein and NO production. These ingredients are also able to suppress the activation of NF-kB, which is activated by TNF-α (Pozniak et al., 2014) and triggers the activation of IL-6 (Hämäläinen et al., 2007). In addition, hesperidin has been proven to be beneficial against COVID-19 due to its immunomodulatory effects and antiviral activities, which result in inhibition of SARS-CoV-2 M^pro^ (Ngwa et al., 2020). Quercetin is another flavonoid with an antiviral effect against SARS-CoV-2 M^pro^ (Sharma et al., 2022). Hence, Virofree, which consists of a combination of flavonoids with anti-inflammatory and antiviral properties, may have potential as a candidate herbal medicine against COVID-19.

In conclusion, the data suggest that Virofree can target various stages of viral entry and replication, especially against Delta and Omicron variants, as well as the following consequences including ferroptosis, cytokine storm, ARDS, and pulmonary fibrosis (Figure 7). These findings emphasized the potential of Virofree as a multiple-function herbal medicine to reduce SARS-CoV-2 infection in the circumstance that there are no specific and effective drugs for new variants and to alleviate post-infection complications.

## Data Availability

The original contributions presented in the study are included in the article/Supplementary Material, further inquiries can be directed to the corresponding authors.
