# Supplementary material for: Virofree, an Herbal Medicine-Based Formula, Interrupts the Viral Infection of Delta and Omicron Variants of SARS-CoV-2
Source: Front Pharmacol. 2022 Jul 4;13:905197. doi: 10.3389/fphar.2022.905197 (PMC9289459; doi:10.3389/fphar.2022.905197)
Supplement: Supplementary file 1 [file DataSheet1.docx]

**Supplementary information**

**Supplementary Table 1. Enrichment analysis of Virofree treatment transcriptomic profiles in gene sets related to COVID-19 pathology.**

**
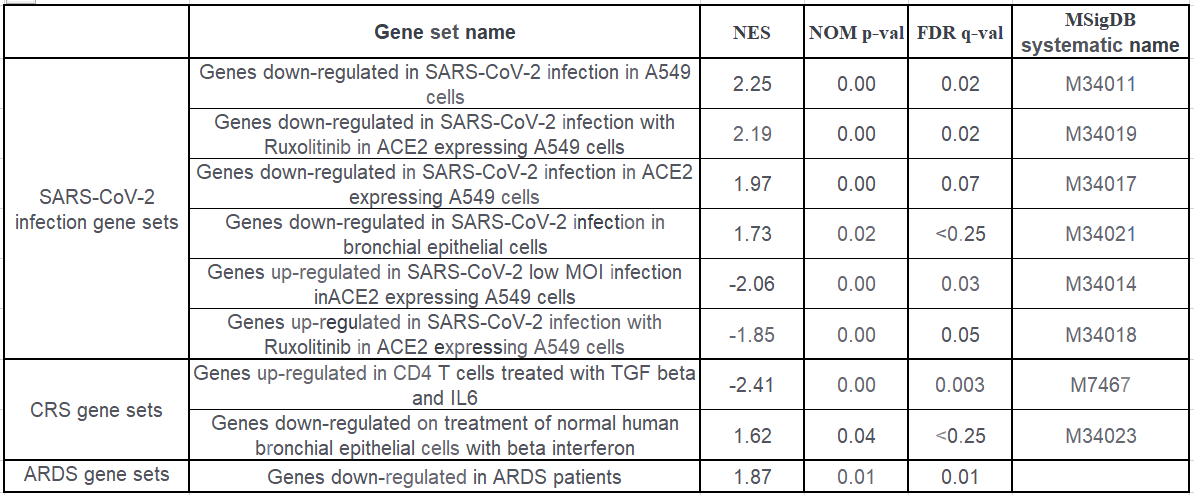
**

*CRS: Cytokine release syndrome, as known as cytokine storm*

**Supplementary Table 2. Significant up and downregulated genes of high and low-dose Virofree treatment response profiles.**

Low dose (66.67 µg/ml) showed 6 up and 8 down of significant differential expression genes. The top 10 DEGs of high dose (500 µg/ml) are listed in the table.


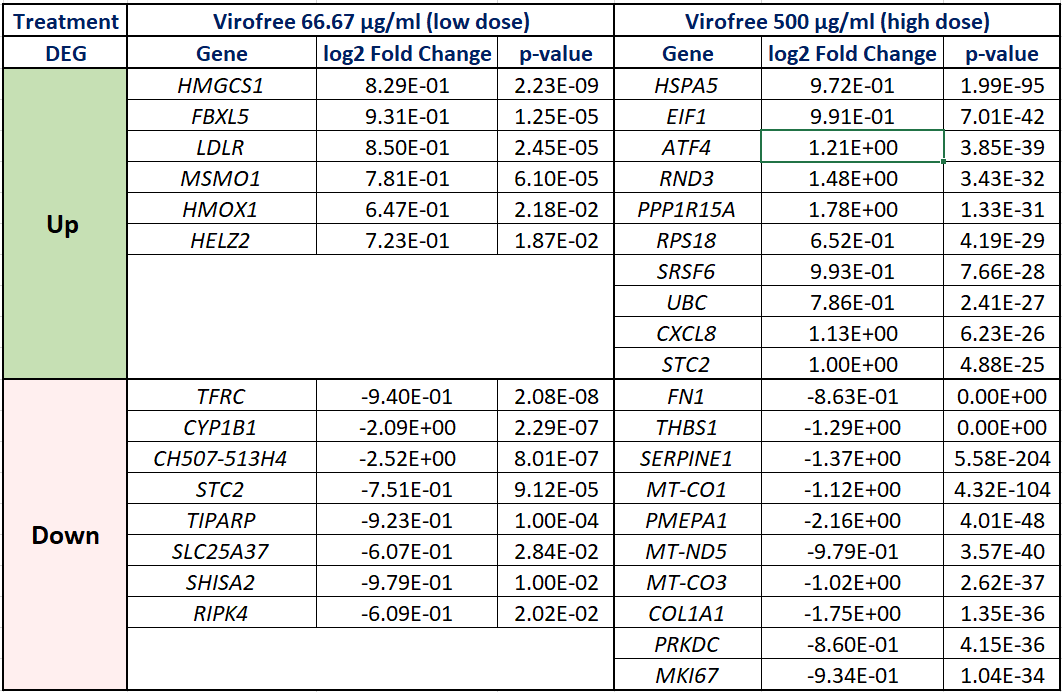


*DEG: differential expression gene.*

**Supplementary Table 3. The detailed list of prediction pathways and diseases of low-dose Virofree from multi-databases.**

**Supplementary Table 4. The detailed list of prediction pathways and diseases of high-dose Virofree from multi-databases.**

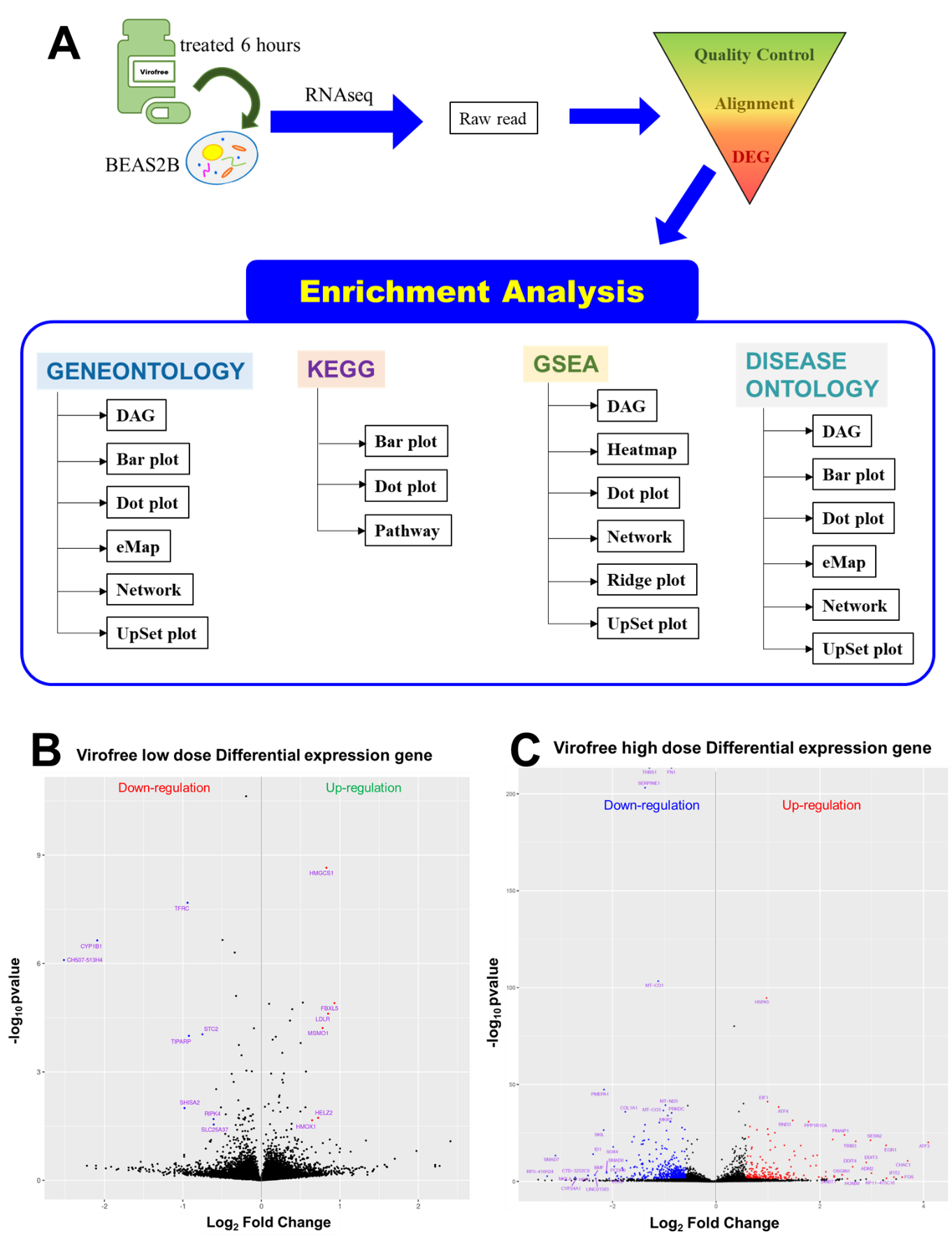


**Supplementary Figure 1. The overview of the analysis pipeline**

(A) After 6 h of 66.67 μg/ml or 500 μg/ml of Virofree treatment in BEAS2B, RNA was extracted for next-generation sequencing and the substantial differential expression levels and enrichment analysis were analyzed. The platform, including four databases, Gene Ontology (GO), KEGG, Disease Ontology (DO), and GSEA, can reveal the mechanisms of Virofree through big data analysis. The identification of the significant differential expression genes of Virofree. BEAS-2B cells were treated with 66.67 μg/ml (B) or 500 μg/ml Virofree (C) and RNA was extracted for next-generation sequencing. Compared with the control and calculated log2 fold change (x-axis), the positive/negative values denoted up/downregulation genes were shown as a volcano plot. The y-axis showed the significant change between Virofree and the control. Cut-off denoted 1.5 log2 fold change. The detailed information is listed in **Supplementary Table 2**.


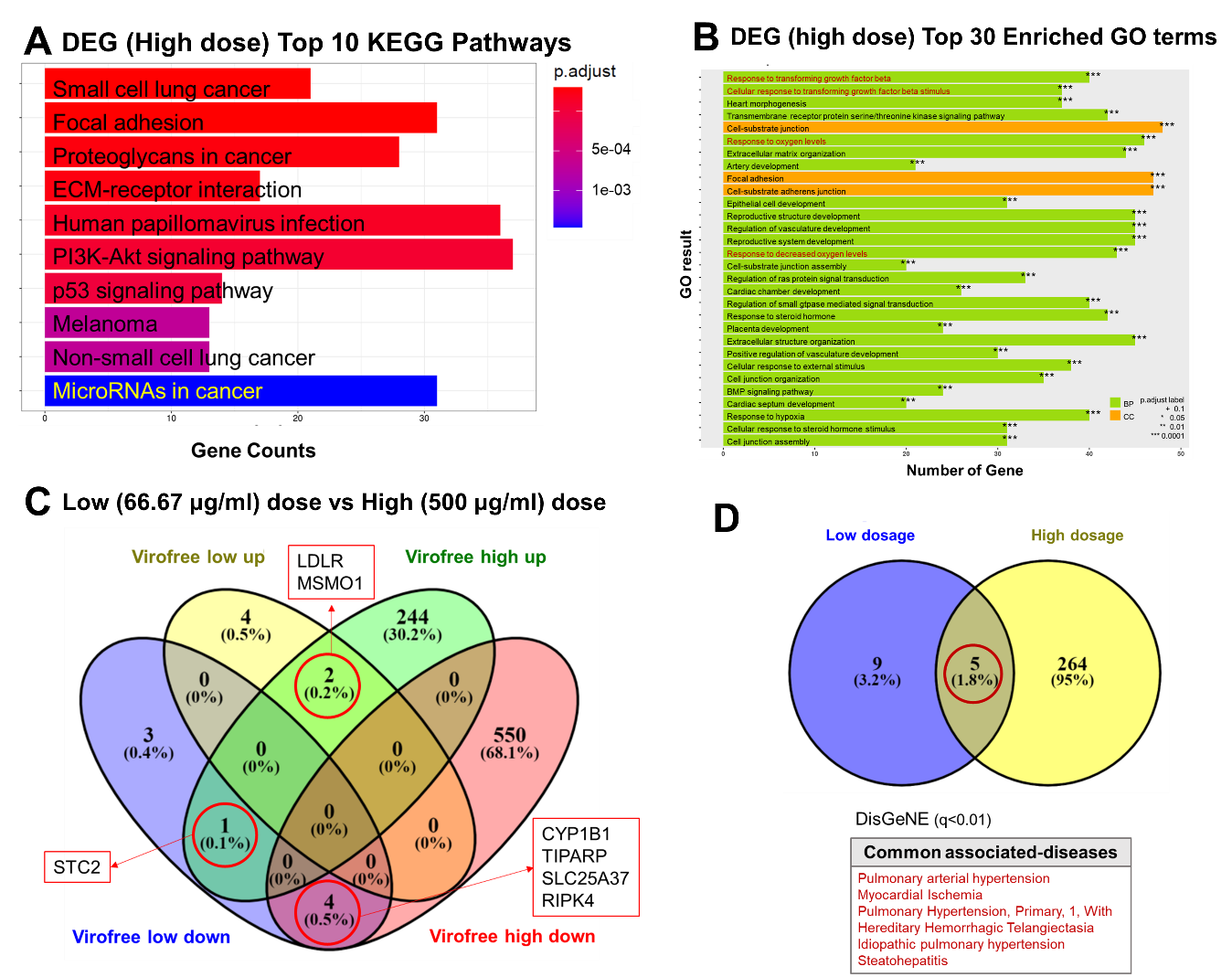


**Supplementary Figure 2. Different dosages of Virofree connected similar mechanisms through bioinformatics analysis.**

The highly potential disease mechanisms of high dose (500 μg/ml) of Virofree from big data analysis. (A) The bar plot showed the top 10 pathways from KEGG enrichment analysis on the DEGs list. The intensity of color denotes the *p*-value, and the length of the bar indicates the overlapped genes ratio (gene counts) between the DEGs and pathways. (B) The bar plot demonstrated the top 30 pathways of high Virofree from Gene Ontology (GO). The color means the classification of the gene, such as Biological Process (BP) and Cellular Component (CC). The length of the bar indicates the overlapped genes number between the DEGs and GO terms. The analysis results were listed in **Supplementary Table 4.** (C-D) The effects on genes expression levels and associated diseases of low dose (66.67 μg/ml) of Virofree were compared with the effects of high dose (500 μg/ml) of Virofree. (C) Venn diagram intersected the common DEGs among 4 sets and 2 genes (LDLR and MSMO1) were up-regulated at both doses as well as 4 genes (CYP1B1, TIPARP, SLC25A37, and RIPK4) were downregulated at both. However, expression levels of STC2 were changed between low and high dosage. The threshold is 1.5 log2 fold change and the q value < 0.01. (D) We intersected the disease results between two concentrations and showed 5 common diseases in both treatments. Especially, pulmonary arterial hypertension was one of the elements that Virofree was the candidate drug to treat ARDS.

**Supplementary Figure 3. Cytotoxicity of Virofree treatments.**

After 24 h of Virofree treatments, BEAS2B cells were collected and measured the survival rate by SRB assay (*n* = 3). All data are presented as means ± SD.

**Supplementary Figure 4. Virofree can potentially reduce viral replication by inducing *let-7a-5p.*** *let-7a-5p* expression levels were measured by qRT-PCR after 24 h treated with 333 µg/ml of Virofree (*n* = 3). All data are presented as means ± SD. Statistical analysis was carried out with a t-test. *: significantly different from the corresponding control respectively with p < 0.05.

**Supplementary Figure 5. The inhibitory activity of Virofree against the Delta RBD spike protein binding to ACE2.**

RBD spike protein derived from variant Delta was used for ELISA-based spike protein and hACE2 binding assays. A positive condition represents the full binding activity of trimeric spike protein on hACE2. The inhibitor used was the wild-type spike RBD antibody (10 μg/mL). Data represent Mean ± SEM (n = 4). A **or *** indicates a significant difference to the corresponding control sample with *p* < 0.01 or 0.001, respectively when compared to the binding efficiency of the positive group.


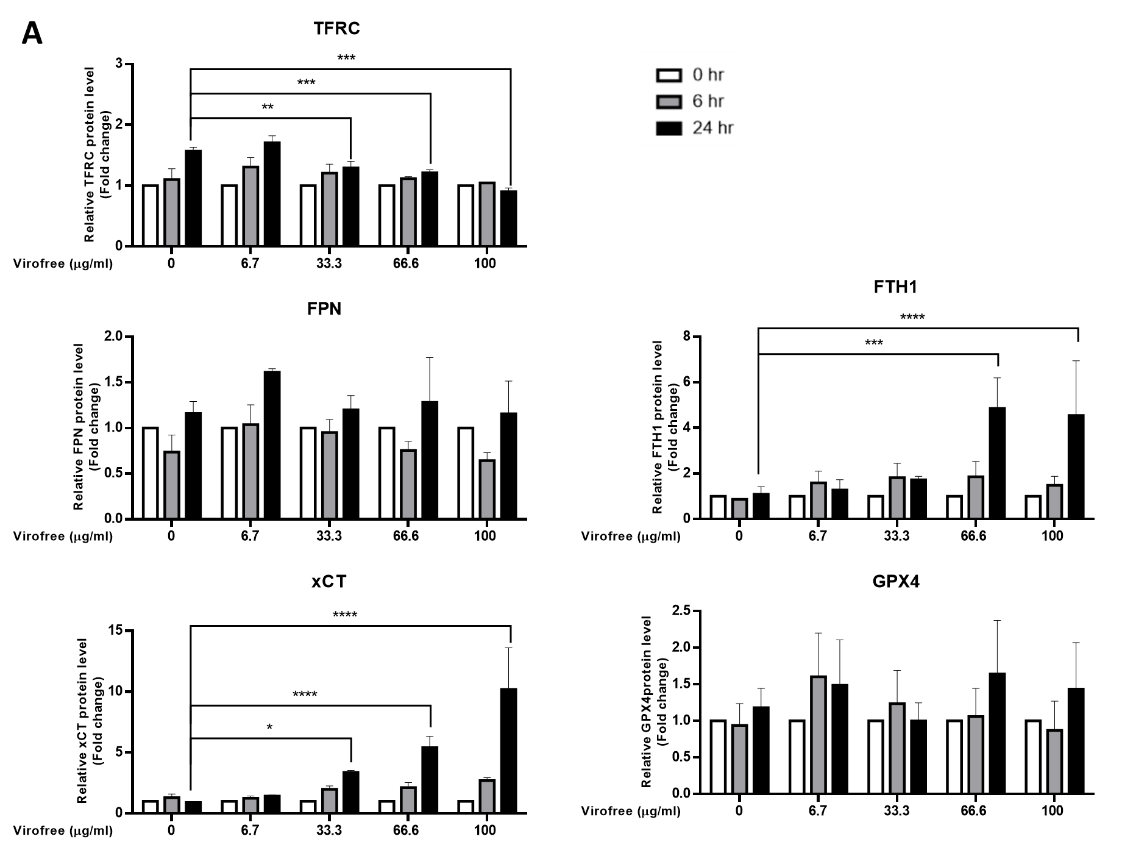


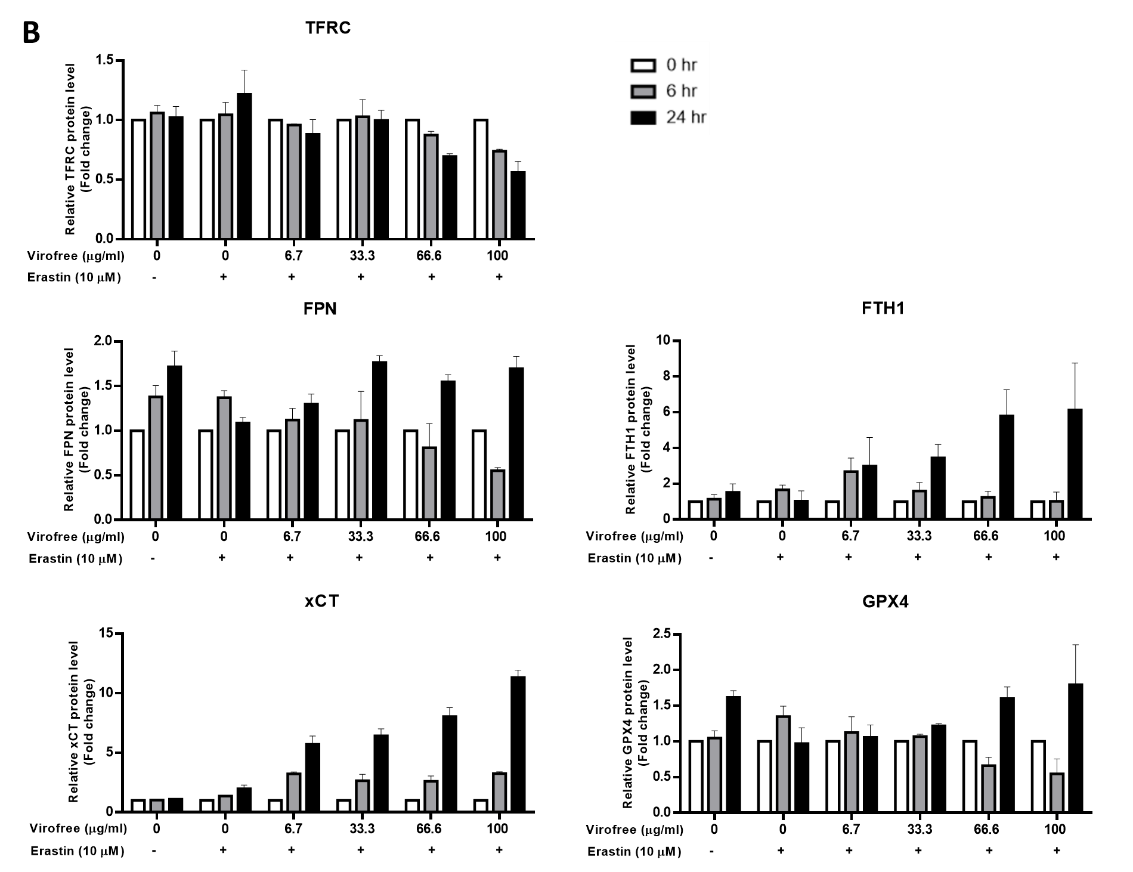


**Supplementary Figure 6. Quantification of Virofree reducing labile iron pool and protecting the cells from ferroptosis in THP-1-derived macrophages.**

(A) THP-1-derived macrophages received treatments of different concentrations of Virofree for 6, or 24 h, respectively. GAPDH was used as an internal control (*n* = 3). (B) THP-1-derived macrophages received treatments of different concentrations of Virofree in the presence of erastin (10 μM) for 6, or 24 h, respectively. GAPDH was used as an internal control (*n* = 3). All data are presented as means ± SD. A *, **, ***, or **** indicates a significant difference to the corresponding control sample with *p* < 0.05, 0.01, 0.001, or 0.0001, respectively.


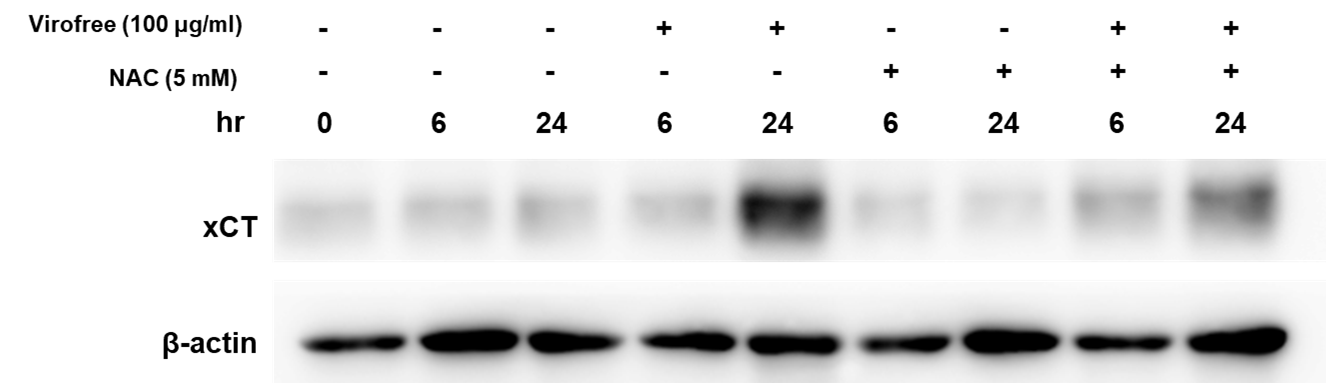


**Supplementary Figure 7. The reduction of ROS levels mediated by NAC decreased the expression levels of xCT mediated by Virofree.**

THP-1-derived macrophages were pretreated with 5 mM NAC for 1 h and then treated with 100 μg/ml of Virofree for 6, or 24 h, respectively. Whole-cell lysates were prepared and subjected to western blot analysis. β-actin was used as an internal control.


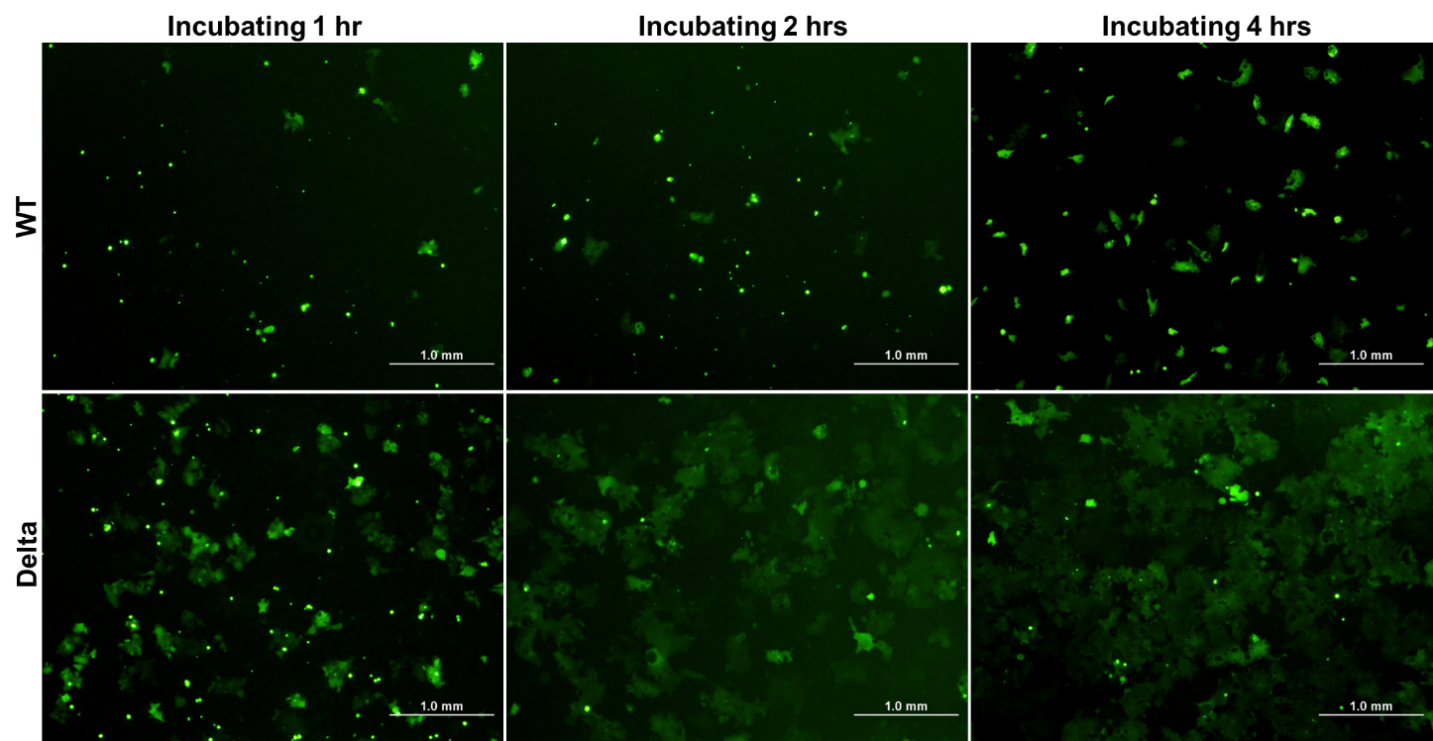


**Supplementary Figure 8. Time-course assay for syncytium formation of both wild-type and Delta spike-expressed BHK21 to ACE2-expressed Calu-3 cells**

EGFP and wild-type (upper panel) or Delta (lower panel) Spike Co-expressed BHK21 cells were added into Calu-3 cells and incubated at 37^o^C for 1, 2, or 4 h. The big fluorescence multinucleate cells were formed in the control group, indicating spike-mediated syncytium formation


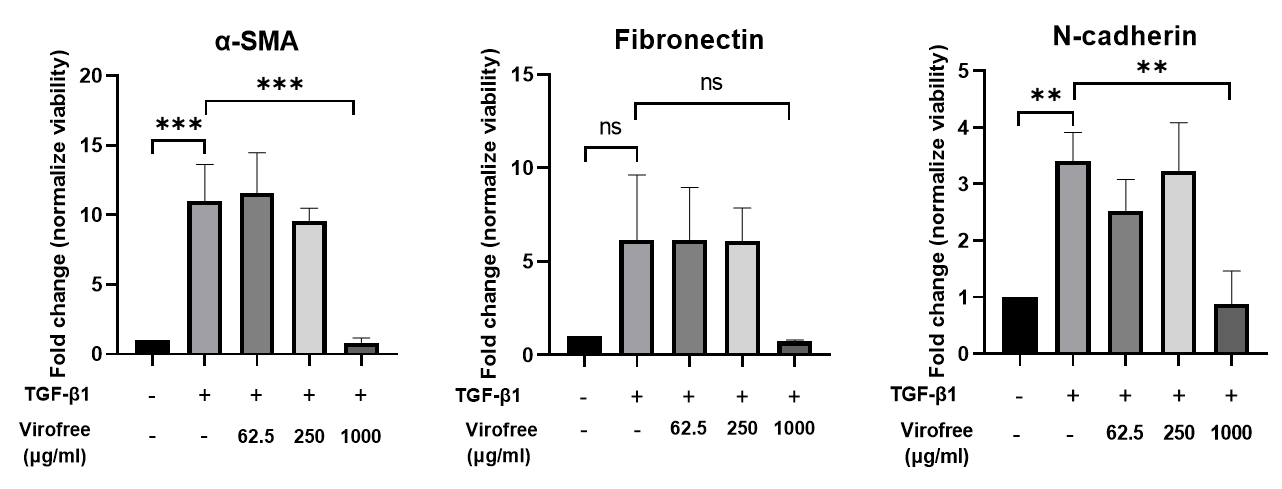


**Supplementary Figure 9. Quantification and statistical analysis of Western blot shown in Figure 6.**


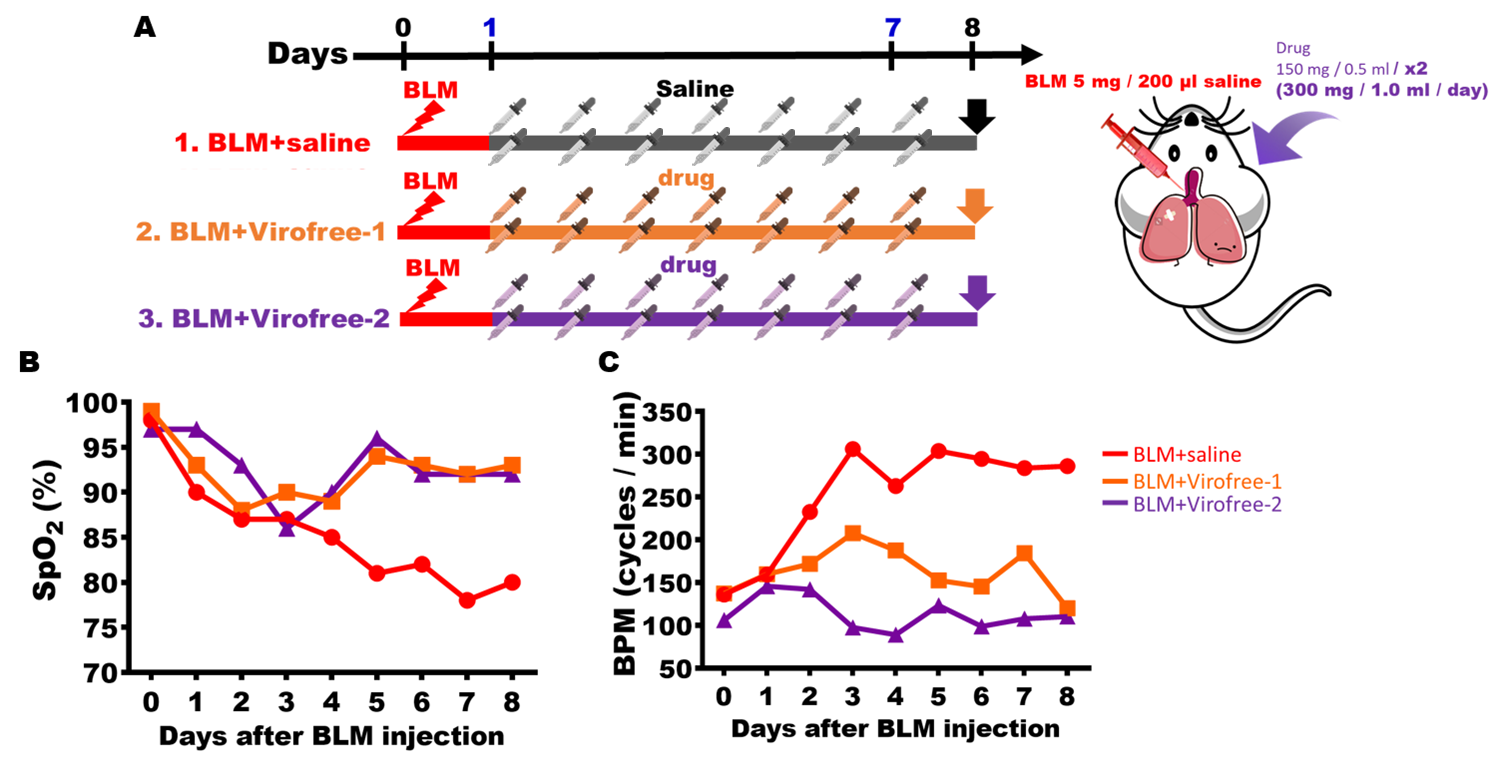


**Supplementary Figure 10. Virofree improved pulmonary function in BLM-induced ARDS rats.**

(A) Experimental scheme of Virofree treatment; (B) Arterial blood oxygen saturation (SpO_2_) and (C) Respiratory rate (BPM: Breath per min)

**Supplementary Figure 11. Cytotoxicity of Virofree treatment on differentiated THP-1 cells.**

PMA-differentiated THP-1 cells were treated with Virofree, with or without LPS 100 ng/ml. After 24 h, an MTS assay was performed to measure cell viability. The group without any treatment was considered a control group. *(n=3)*


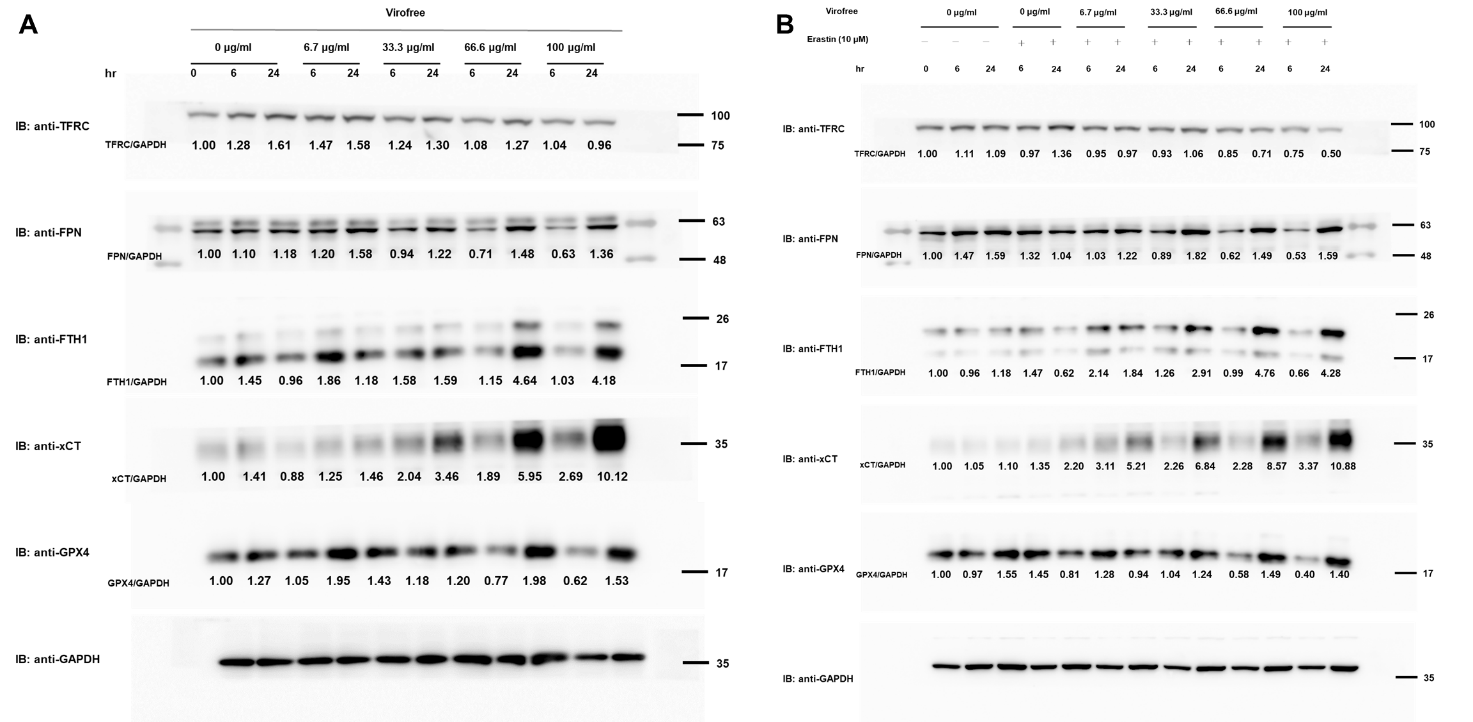


**Supplementary Figure 12. Whole un-cropped images of the original Western blots shown in Figure 5.**


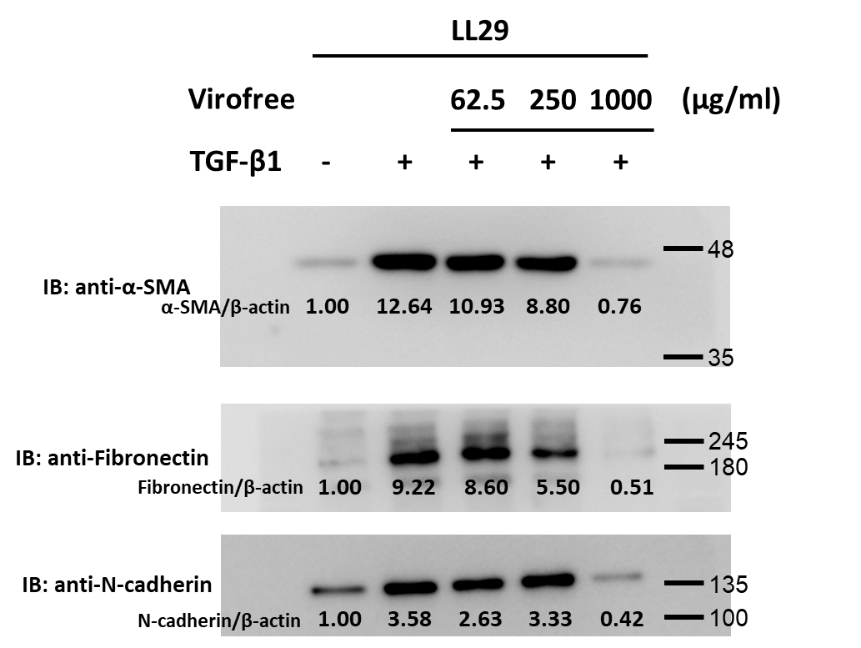


**Supplementary Figure 13. Whole un-cropped images of the original Western blots shown in Figure 6.**
